# Cost‐Effectiveness of a Personalised Self‐Management Intervention for People Living With Long Covid: The LISTEN Randomised Controlled Trial

**DOI:** 10.1111/hex.70357

**Published:** 2025-08-04

**Authors:** Shaun R. S. Harris, Bernadette Sewell, Monica Busse‐Morris, Adrian Edwards, Fiona Jones, Fiona Leggat, Philip Pallman, Deborah Fitzsimmons

**Affiliations:** ^1^ Swansea Trials Unit Swansea University Swansea UK; ^2^ Swansea Centre for Health Economics Swansea University Swansea UK; ^3^ Centre for Trials Research, School of Medicine Cardiff University Cardiff UK; ^4^ PRIME Centre Wales, Division of Population Medicine, School of Medicine Cardiff University Cardiff UK; ^5^ Wales COVID‐19 Evidence Centre Cardiff UK; ^6^ Population Health Research Institute, St George's University of London London UK; ^7^ Kingston University London London UK; ^8^ Bridges Self‐Management London UK

**Keywords:** cost‐effectiveness, Covid‐19, long Covid, rehabilitation, self‐management

## Abstract

**Background:**

In the United Kingdom, at least 1.9 million people are estimated to have experienced long Covid, of which 1.3 million have symptoms lasting for more than a year. The Long CovId Personalised Self‐managemenT support EvaluatioN (LISTEN) trial evaluated the effectiveness and cost‐effectiveness of a co‐designed personalised self‐management support intervention for non‐hospitalised people living with long Covid.

**Methods:**

We conducted a pragmatic, multicentre, two‐arm, parallel group and superiority randomised controlled trial for people who had experienced at least one long Covid symptom for 12 weeks or longer. A cost–utility analysis was undertaken alongside the LISTEN trial from both a UK National Health Service (NHS) and personal social services (PSS) and a societal perspective. Implementation costs were determined from study records, and quality of life and health and care resource use were collected by questionnaire at 6‐week and 3‐month follow‐ups. Incremental net monetary benefit (INMB) analyses evaluated the cost‐effectiveness of the intervention at a range of willingness‐to‐pay thresholds.

**Results:**

A total of 544 participants were included in the health economic analysis, of which 62.5% had complete data. The average cost of delivering the LISTEN intervention was £846 per participant. At 3‐month follow‐up, mean quality‐adjusted life years (QALYs) were 0.005 (95% CI −0.004 to 0.014) greater for participants receiving the LISTEN intervention compared to usual care. From the NHS and PSS perspective, total adjusted mean costs were £491 (95% CI, £128 to £854) lower in the usual care arm. From the societal perspective, participants in the usual care arm lost more hours of work and usual activities and received more informal care, with the LISTEN intervention dominating usual care.

**Conclusions:**

At accepted UK thresholds, the LISTEN intervention was not cost‐effective from an NHS and PSS perspective, but it was found to be cost‐effective from a societal perspective due to the impact of long Covid on work, informal care and usual activities. Further research is required to understand the costs and benefits of self‐management support for longer‐term horizons.

**Patient and Public Contribution:**

We are grateful for the contributions of the LISTEN Public and Patient Involvement and Engagement group comprising seven people (Anne Domeney, Ian Patel, Carol Rowe, Judith Parsons, Rebecca Beltran, Elizabeth Treadwell and Maria Ines de Sousa de Abreu) with long Covid who supported co‐design, communications, trial recruitment and dissemination activities.

**Trial Registration:**

ISRCTN36407216, registered 27 January 2022.

## 1 Introduction

Many people experiencing Covid‐19 make a full recovery within a few weeks after their first symptoms. However, some people have longer‐lasting symptoms. These long‐term effects have been described by the World Health Organization (WHO) as long Covid. The condition is defined as the continuation or development of new symptoms 3 months after initial infection, with these symptoms lasting for at least 2 months with no other explanation [1]. Symptoms are often persistent and fluctuating and include cough, shortness of breath and chest pain [1, 2].

In the United Kingdom, at least 1.9 million people are estimated to meet the criteria for long Covid. Of these, 1.3 million have symptoms lasting for more than a year, and 762,000 people live with symptoms for more than 2 years, with many experiencing reductions in quality of life [3]. Lived experiences have been found to vary, with some people feeling well‐supported whilst others have difficulty accessing and navigating services [4].

The impact of long Covid is not confined to the individual or health service. Wider economic costs from the loss of work and informal care also need to be considered. The potential for a legacy of long Covid is serious, with approximately 80,000 people in the United Kingdom estimated to have left the workforce due to long Covid [5], and others reducing hours or modifying duties when returning to work, and continuing to experience limitations in their day‐to‐day activities [6, 7, 8]. Estimates suggest the impact of long Covid may result in macroeconomic costs of £1.5bn each year to the United Kingdom [9] and more widely, a 1% reduction in global economic activity [10].

The Long CovId Personalised Self‐managemenT support EvaluatioN (LISTEN) trial evaluated the effectiveness and cost‐effectiveness of a co‐designed personalised self‐management support intervention for non‐hospitalised people living with long Covid. Underpinned by social cognitive theory and self‐efficacy principles to build belief in individual capability, the LISTEN intervention drew upon theory and evidence from Bridges Self‐Management to enhance the knowledge, skills and confidence of people to manage everyday life with symptoms of long Covid [11]. The main findings of the trial and the process evaluation were that the intervention resulted in short‐term improvements in routine activities, emotional well‐being, fatigue, quality of life and self‐efficacy in people living with long Covid [12].

The trial included an embedded process evaluation to inform understanding of trial processes and implementation and a health economic evaluation considering the within‐trial cost‐effectiveness of the LISTEN intervention [13]. The REGAIN trial [14] recently published evidence that an online, home‐based group physical and mental health rehabilitation intervention was likely to be cost‐effective for adults with long Covid who were initially admitted to hospital. Evidence of other effective treatments for people with long Covid is gradually emerging [15, 16, 17, 18]. Supporting evidence of cost‐effectiveness would help inform decision‐makers to determine which services to offer to ease the burden associated with this condition.

The aim of the health economic analysis was to assess the cost‐effectiveness of the LISTEN self‐management support intervention for people with long Covid using cost and outcome data collected in the LISTEN trial.

## 2 Methods

### 2.1 Trial Design and Patient Population

The LISTEN trial was a pragmatic, multicentre, two‐arm, parallel group and superiority trial involving participants who had experienced at least one long Covid symptom for 12 weeks or longer. Participants were recruited between June 2022 and November 2023 from 15 primary and secondary care National Health Service (NHS) centres and one non‐NHS site within England and Wales. People who were hospitalised for treatment of symptoms during the acute phase of Covid‐19 were not eligible for the trial.

Participants were individually randomised to either the LISTEN intervention or the usual care study arm. The LISTEN intervention consisted of up to 6 one‐to‐one personalised self‐management sessions with a trained LISTEN practitioner and an accompanying co‐designed handbook with a focus on supporting the knowledge, skills and confidence of people with symptoms of long Covid to manage everyday life. Usual care consisted of NHS routine care as available to participants in their region. People in the intervention arm were still allowed to access usual medical care but could not receive rehabilitation. This varied considerably across England and Wales, ranging from no access to mobile applications and resources, and specialist long Covid clinics as detailed in the process evaluation [12]. Full eligibility criteria, details of the intervention, the trial protocol and main results are published elsewhere [13, 19]. Eligible participants provided informed consent electronically.

### 2.2 Economic Analysis

The health economic analysis plan (available as Supporting Material) was finalised before the senior authors having sight of the data. The health economic evaluation was undertaken from two different perspectives. A UK NHS and personal social services (PSS) perspective was adopted in the first instance, in line with National Institute for Health and Care Excellence (NICE) methodological recommendations [20, 21]. However, to allow a detailed analysis of the true cost of long Covid, the cost impact on patients, carers and society (e.g., through time off work and productivity losses) was included in a separate societal analysis. A time horizon of 3 months was used based on trial follow‐up. Due to the lack of available evidence, decision analytic modelling for the longer‐term cost‐effectiveness of the LISTEN intervention was beyond the scope of this study. No discounting of costs or outcomes was applied as the time horizon of the analysis did not exceed 1 year. Conduct and reporting followed Consolidated Health Economic Evaluating Reporting Standards (CHEERS) [22] guidelines.

### 2.3 Estimating the LISTEN Intervention Implementation and Delivery Costs

The LISTEN intervention cost (including intervention co‐design and development, practitioner training and support, and resources required for intervention delivery) was estimated through review of trial and practitioner notes, co‐design activity and training logs, and discussions with the study team.

Co‐design and intervention development included staff time for recruitment of co‐design participants, preparing, organising and running co‐design meetings, undertaking narrative interviews, analysing surveys, developing and refining training materials, writing and editing of the LISTEN book, as well as practitioner time spent on meetings and with participants for survey completion [11]. Co‐design and development costs were considered ‘sunk’ costs and were mainly accrued by non‐NHS staff. As such, these costs were reported separately and not included in the overall LISTEN intervention implementation cost.

Practitioner training and support comprised initial training sessions (either group‐based or one‐on‐one) provided in seven training blocks, and the development and running of a novel wraparound support package which included weekly to bi‐weekly question and answer (Q&A) sessions, one‐to‐one advice, written and video resources, and psychological support. Required staff time included training session organisation, preparation and delivery, as well as communications and the time of participating practitioners. Every practitioner also received a copy of the LISTEN book. The process for intervention delivery was costed using the average session time across all sessions delivered and the total number of sessions for each participant to calculate the cost of sessions for each participant based on the profession and salary band of the delivering practitioner. Where no salary bands were available, the average pay band across practitioners where pay band information was available was used. Session preparation and compilation of practitioner notes before and after each session were assumed to require 30 min each as recommended in the intervention guidance. An estimate for printing and postage costs for the LISTEN book for English and Welsh versions was included. The cost per participant was calculated by dividing the total cost of the LISTEN intervention by the number of participants who had received at least one session of the LISTEN intervention.

### 2.4 Estimation of Health and Care Resource Use Costs

Health service resource use was established using an adapted Client Service Receipt Inventory (CSRI) questionnaire [23]. The CSRI was adapted for long Covid with support from the trial team, relevant clinicians and public members of the co‐design team living with long Covid to provide an appropriate overview of the burden of long Covid to the NHS, patients and society. The CSRI was completed by participants at baseline and the 6‐week and 3‐month follow‐up points. Relevant healthcare resources included primary care and secondary care attendances, social and community‐based care, as well as tests and investigations. Participant‐ and society‐borne costs such as loss of earnings, participant out‐of‐pocket expenses (e.g., private healthcare appointments) and opportunity costs incurred by family members providing informal care were used for the societal perspective. Unit costs for health resources were obtained from standard published unit costs [24, 25, 26] and inflated to 2022/23 prices using the NHS cost inflation index (NHSCII) [24]. Opportunity costs associated with lost work, normal activities and usual care were valued using median hourly earnings based on 37 total weekly paid hours as reported by the Office of National Statistics [27].

### 2.5 Health‐Related Quality of Life and Other Health Outcomes

The primary health economic outcome for the cost–utility analysis (CUA) was the quality‐adjusted life year (QALY), as recommended by NICE [28], derived from the EuroQol 5‐Dimension 5‐Level (EQ‐5D‐5L) questionnaire completed by participants at baseline and the 6‐week and 3‐month follow‐up points. Utility values were estimated using the validated mapping function to existing EQ‐5D‐3L UK utility tariffs [29]. Quality of life preference scores derived from the EQ‐5D visual analogue scale (VAS) were converted to a 0–1 scale. QALYs from the EQ‐5D descriptive system were calculated by linear interpolation using the area‐under‐the‐curve approach [30].

### 2.6 Analysis

Comparative analysis to establish differences between groups was based on the intention‐to‐treat (ITT) population. A total cost per participant was calculated by combining healthcare resource use with relevant unit costs. Mean resource usage and 95% confidence intervals (CIs) were calculated based on all available cases and tabulated for both intervention and usual care groups at each data collection point. CIs for count variables (e.g., number of contacts) were calculated from the Poisson distribution.

Assuming data were missing at random, multiple imputation by chained equations using predictive mean matching was used to address missing cost and outcome data. The imputation model included age, sex, trial site, qualifications and employment status as covariates. Rubin's rules [31] were used to combine the 67 imputed datasets equivalent to the percentage of participants with complete data, and a randomisation seed was used to enable reproducible imputations.

The primary analysis consisted of a CUA undertaken to calculate the cost per QALY gained of the LISTEN intervention compared to usual care. Mean costs and QALYs by trial group and their differences were estimated using seemingly unrelated regression, accounting for the correlation between costs and QALYs [32, 33]. Costs and QALYs were adjusted for baseline costs, baseline EQ‐5D‐5L utility values and site consistency. There was no discounting of costs or effects given the 3‐month trial duration.

Net monetary benefits (NMBs) were calculated for willingness‐to‐pay thresholds between £0 to £100,000 per QALY. Cost‐effectiveness acceptability curves (CEACs) were generated to explore sample uncertainty and estimate the probability that the LISTEN intervention was more cost‐effective than usual care across a range of willingness‐to‐pay thresholds.

A cost‐effectiveness analysis (CEA) compared the mean adjusted difference in the routine activities domain score of the Oxford Participation and Activities Questionnaire (Ox‐PAQ) [34], the primary clinical effectiveness outcome between the LISTEN intervention and usual care (the incremental effect), to the incremental cost. The resulting incremental cost‐effectiveness ratio (ICER) was expressed as the incremental cost per point improvement in the Ox‐PAQ routine activities domain score.

A descriptive cost–consequences analysis (CCA) approach reported the full range of disaggregated costs and primary and secondary outcomes of the trial in tabulated form to provide a transparent, comprehensive and easily accessible summary of the trial results, which can be particularly useful for decision‐makers.

One‐way sensitivity analyses were performed to address parameter uncertainty. A scenario analysis was also undertaken that considered the cost‐effectiveness of the LISTEN intervention within a routine health and care setting, accounting for potential economies of scale affecting the LISTEN intervention cost that could not be observed in the short time horizon of the trial.

A detailed health economic analysis plan was finalised before analysis, which was conducted using Stata version 17.0.

## 3 Results

### 3.1 LISTEN Intervention Implementation Cost

Co‐design of the LISTEN intervention with practitioners and people living with long Covid, as well as intervention material and practitioner training development, and development and design of the LISTEN handbook, accrued a total cost of £27,742. These development costs were not included in the total LISTEN intervention implementation costs.

Practitioner training (including 59 practitioners in group sessions and 11 in individual sessions) and ongoing support (Q&A sessions, one‐on‐one sessions and psychological support) amounted to £64,461, or £921 per practitioner trained. During the study period, LISTEN practitioners delivered 1256 LISTEN intervention sessions to 237 participants who received at least one session and attended an average of 5.30 sessions (SD 1.47). The average session duration was 56.11 min (SD 9.84) at an overall intervention delivery cost of £133,149. Including the LISTEN handbook at a cost of £11.09 per handbook, the total LISTEN intervention cost was £200,603, or £846 per participant who received at least one session of the intervention (*n* = 237).

### 3.2 Health Economic Analysis

Resource use and cost data were available for 544 participants. Participants had a median age of 50 years, 72% were female, and 92% were white. Across both groups, they had experienced a median of 12 different symptoms related to their long Covid, and 88% had received a positive Covid‐19 test. The proportion of participants returning complete EQ‐5D and CSRI questionnaires was 62.5%.

Table 1 presents the observed EQ‐5D‐5L VAS and utilities for the LISTEN intervention and usual care treatment groups for baseline and each follow‐up. EQ‐5D‐5L scores at 6‐week and 3‐month follow‐ups were slightly higher in the LISTEN group, but the difference was not statistically significant. Analysis of individual EQ‐5D dimensions showed that floor effects are unlikely to be a major factor, with the proportion of participants providing the lowest score on each dimension not exceeding an established 15% threshold [35].

**Table 1:** Mean utility scores for each group.

	LISTEN intervention *N* = 270		Usual care *N* = 274	
Time point	*n*	EQ‐5D‐5L utility (95% CI)	*n*	EQ‐5D‐5L utility (95% CI)
Baseline	268	0.50 (0.46, 0.54)	273	0.53 (0.49, 0.56)
6 weeks	209	0.54 (0.50, 0.57)	222	0.51 (0.47, 0.55)
3 months	208	0.54 (0.50, 0.58)	196	0.53 (0.49, 0.57)
	***n***	**EQ‐5D VAS (95% CI)**	***n***	**EQ‐5D VAS (95% CI)**
Baseline	265	44.95 (42.44, 47.46)	266	45.74 (43.22, 48.26)
6 weeks	209	51.14 (48.40, 53.89)	221	49.29 (46.30, 52.28)
3 months	206	49.66 (46.70, 52.62)	190	48.36 (44.97, 51.76)

Abbreviations: CI, confidence interval; EQ‐5D‐5L, EuroQol 5‐Dimension 5‐Level Questionnaire; VAS, visual analogue scale.

The cost associated with health and care resource use for participants in both trial groups is summarised in Supporting Appendix. The mean number of contacts across both arms was typically small. The most frequently used resource items were outpatient visits and long Covid rehabilitation programme sessions.

A summary of resource use costs (Table 2) shows that at both 6‐week and 3‐month follow‐ups, participants in the LISTEN intervention group had lower costs across healthcare areas, including primary/community care, secondary care and medications.

**Table 2:** Summary of health and care and societal costs for people living with long Covid in the LISTEN intervention group compared to the usual care group.

	Time point	LISTEN intervention		Usual care	
		*n*	Total costs (£) (95% CI)	*n*	Total costs (£) (95% CI)
Primary/community care	Baseline	244	201.66 (143.94, 259.38)	250	197.90 (137.26, 258.55)
Primary/community care	6 weeks	184	63.45 (29.59, 97.32)	194	139.65 (81.32, 197.98)
Primary/community care	3 months	174	52.48 (21.68, 83.29)	154	140.69 (75.65, 205.73)
Secondary care	Baseline	235	233.95 (189.23, 288.67)	245	225.78 (173.05, 278.51)
Secondary care	6 weeks	196	75.58 (48.97, 102.19)	203	103.13 (73.73, 132.52)
Secondary care	3 months	169	113.02 (61.03, 165.00)	145	153.63 (95.31, 211.95)
Tests	Baseline	250	109.88 (82.14, 137.63)	260	102.40 (74.81, 129.99)
Tests	6 weeks	204	61.85 (−24.05, 147.76)	217	34.07 (17.61, 50.52)
Tests	3 months	189	47.69 (24.02, 71.35)	181	57.34 (25.86, 88.83)
Mental health	Baseline	259	20.08 (10.09, 30.08)	262	26.11 (10.54, 41.68)
Mental health	6 weeks	197	5.66 (0.28, 11.05)	209	8.36 (2.81, 13.91)
Mental health	3 months	190	7.66 (2.04, 13.28)	180	19.42 (3.87, 34.97)
Medications	Baseline	264	41.46 (6.894, 76.08)	272	22.33 (9.24, 35.43)
Medications	6 weeks	209	10.86 (6.87, 14.85)	216	15.75 (5.98, 25.52)
Medications	3 months	121	17.32 (10.19, 24.46)	180	18.67 (5.11, 32.23)
Societal costs					
Lost work/usual activity costs	Baseline	233	3609.33 (3079.29, 4139.36)	249	4127.42 (3525.57, 4729.28)
Lost work/usual activity costs	6 weeks	191	1784.56 (1500.36, 2068.75)	191	2064.84 (1713.28, 2416.40)
Lost work/usual activity costs	3 months	172	1589.74 (1287.91, 1891.57)	159	2185.97 (1781.43, 2590.51)
Private healthcare costs	Baseline	164	323.85 (23.82, 623.89)	179	185.80 (141.85, 229.75)
Private healthcare costs	6 weeks	128	102.09 (42.46, 161.72)	125	174.11 (92.60, 255.63)
Private healthcare costs	3 months	103	89.85 (53.35, 126.35)	103	182.51 (53.11, 311.91)
Travel costs	Baseline	166	33.93 (15.58, 52.29)	184	36.39 (19.81, 52.98)
Travel costs	6 weeks	153	6.08 (2.52, 9.64)	136	6.72 (3.36, 10.08)
Travel costs	3 months	138	12.77 (5.83, 19.71)	102	11.97 (4.56, 19.38)

At 3‐month follow‐up, participants in the usual care arm reported significantly more private health services and private mental health contacts. For both arms, the largest proportion of societal costs was attributable to lost work and lost time from normal activities, demonstrating the considerable burden of long Covid to both individuals and society.

### 3.3 Cost‐Effectiveness

From an NHS/PSS perspective, the adjusted mean total cost per patient for the LISTEN group was £1870 (95% CI, £1608 to £2131), while for the usual care group, it was £1379 (95% CI, £1115 to £1642) (Table 3). The mean cost difference between the groups shows the LISTEN intervention was £491 (95% CI, £128 to £854) more expensive than usual care. When including patient and societal costs, the adjusted mean total costs for the LISTEN intervention group of £6448 (95% CI, £5719 to £7177) were lower compared to usual care (£7054; 95% CI, £6253 to £7856), demonstrating a cost reduction of £607 (95% CI, −£1690 to £477).

**Table 3:** Cost‐effectiveness results.

Allocation arm	*n*	Mean adjusted cost (£) (95% CI)	Mean adjusted QALY (95% CI)	Incremental cost (£) (95% CI)	Incremental QALY (95% CI)	NMB (£) at £20,000/QALY (95% CI)	NMB (£) at £30,000/QALY (95% CI)
NHS and PSS perspective							
Usual care	274	1378.58 (1114.89, 1642.27)	0.126 (0.120, 0.132)				
LISTEN intervention	270	1869.57 (1608.18, 2130.96)	0.131 (0.125, 0.137)	490.99 (127.97, 854.00)	0.005 (−0.004, 0.014)	−377.59 (−800.14, 44.95)	−322.24 (−789.88, 145.39)
Societal perspective							
Usual care	274	7054.38 (6252.66, 7856.10)	0.126 (0.120, 0.132)				
LISTEN intervention	270	6,447.85 (5719.21, 7176.50)	0.131 (0.125, 0.137)	−606.53 (−1690.13, 477.07)	0.005 (−0.004, 0.014)	1058.48 (−174.88, 2291.84)	1113.83 (−144.32, 2371.98)

*Note:* Results from the multiply imputed dataset. All models are adjusted for site. QALYs are adjusted for baseline utility score. Cost differences are adjusted for the perspective total baseline costs.

Abbreviations: CI, confidence interval; NHS, National Health Service; PSS, personal social services; QALY, quality‐adjusted life year derived from EQ‐5D‐5L questionnaire responses.

Participants in the LISTEN intervention group accumulated slightly more QALYs (mean 0.131; 95% CI, 0.124 to 0.137) than people receiving usual care (mean 0.126; 95% CI, 0.120 to 0.132), with an adjusted mean difference of 0.005 QALYs (95% CI, −0.004 to 0.014). Therefore, from an NHS/PSS perspective, the LISTEN intervention is estimated to have a higher mean cost but also generates more QALYs than usual care, with an ICER of £95,304 per QALY gained. From the societal perspective, the LISTEN intervention dominates usual care. Incremental costs and QALYs are presented in Table 3.

**Figure 1:**
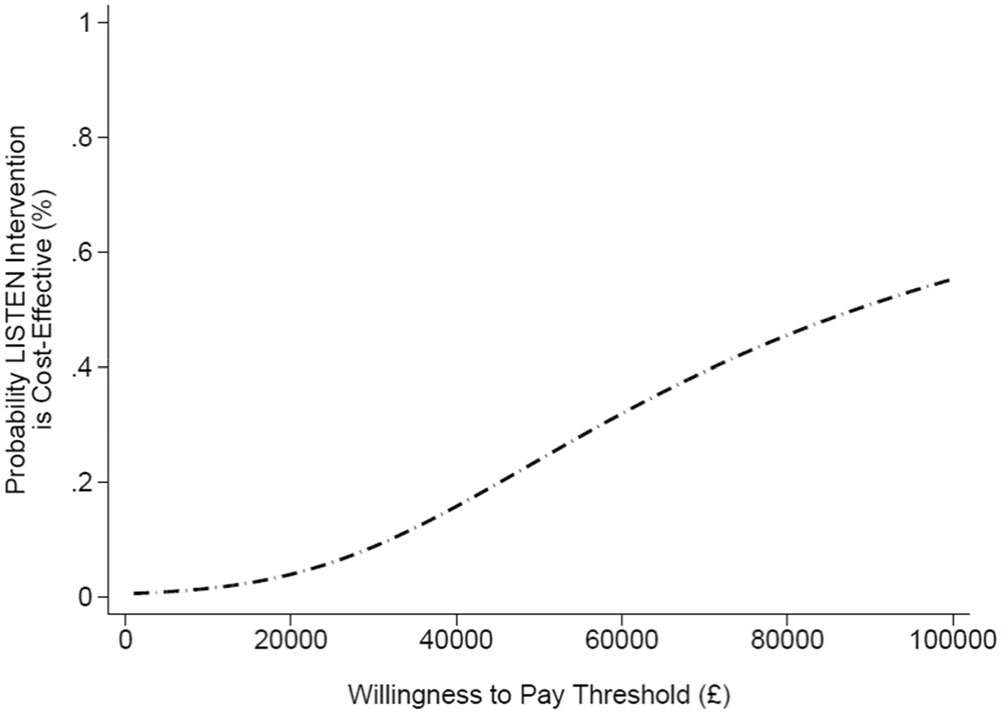
Cost‐effectiveness acceptability curve (NHS/PSS perspective).

Incremental NMBs are presented in Table 3. For the NHS/PSS perspective, the incremental NMB of the LISTEN intervention compared with usual care was −£378 (95% CI, −£800 to £45) at the willingness‐to‐pay threshold of £20,000 per QALY and −£322 (95% CI, −£790 to £145) at the £30,000 threshold. Negative NMB values indicate that the costs to derive the observed QALY gains are greater than the willingness‐to‐pay threshold. Estimation of the CEAC, illustrated in Figure 1, revealed that the probability that the LISTEN intervention is cost‐effective relative to usual care was 3.99% at the £20,000 threshold from the NHS/PSS perspective. Sensitivity analysis excluding the costs of various tests (Table 4), which potentially could be double counted, led to similar conclusions as the primary results.

**Table 4:** One‐way sensitivity analysis.

Allocation arm	*n*	Mean adjusted cost (£) (95% CI)	Mean adjusted QALY (95% CI)	Incremental cost (£) (95% CI)	Incremental QALY (95% CI)	NMB (£) at £20,000/QALY (95% CI)	CE probability at £20,000 per QALY threshold
1. Excluding test costs (including blood test, chest X‐ray, MRI scan and electrocardiogram), which potentially could be double‐counted. NHS and PSS perspective								
Usual care	274	1239.34 (991.99, 1486.70)	0.126 (0.120, 0.132)				
Intervention	270	1758.65 (1521.13, 1996.16)	0.131 (0.125, 0.137)	519.31 (179.89, 858.72)	0.005 (−0.004, 0.014)	−386.52 (−782.51, 9.47)	2.79%

*Note:* Results from the multiply imputed dataset. All models are adjusted for site. QALYs are adjusted for baseline utility score. Cost differences are adjusted for the perspective total baseline costs.

Abbreviations: CI, confidence interval; NHS, National Health Service; PSS, personal social services; QALY, quality‐adjusted life year.

**Figure 2:**
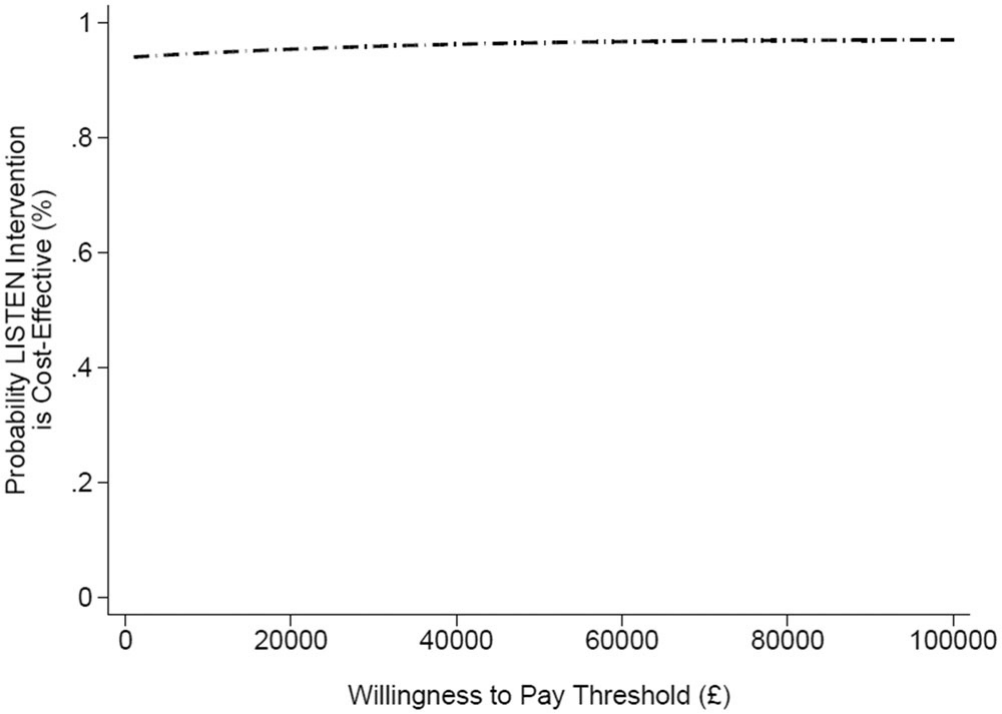
Cost‐effectiveness acceptability curve (societal perspective).

From the societal perspective, the LISTEN intervention dominated usual care, which is reflected in a positive NMB of £1058 (95% CI, −£175 to £2292) and £1114 (95% CI, −£144 to £2372) at thresholds of £20,000 and £30,000 per QALY, respectively. Accordingly, the probability of the LISTEN intervention being cost‐effective compared to usual care at the £20,000 threshold was much higher at 95.40% (Figure 2).

The CEA found positive incremental NMB values for the analysis of cost per point reduction in the Ox‐PAQ routine activities domain score at willingness‐to‐pay thresholds from £200 upwards (Table 5), with large CIs suggesting substantial uncertainty around the point estimate. Whilst no formal willingness‐to‐pay threshold exists for the purposes of decision‐making, the LISTEN intervention can be considered cost‐effective on the Ox‐PAQ routine activities domain score, except for the smallest threshold values.

**Table 5:** Cost‐effectiveness of Ox‐PAQ routine activities domain score, NHS and PSS perspective.

Allocation arm	*n*	Mean adjusted cost (£) (95% CI)	Mean adjusted Ox‐PAQ routine activities domain score (95% CI)	Incremental cost (£) (95% CI)	Incremental Ox‐PAQ routine activities domain score (95% CI)	NMB (£) at £100/point improvement in Ox‐PAQ routine activities domain score (95% CI)	NMB (£) at £200/point improvement in Ox‐PAQ routine activities domain score (95% CI)	NMB (£) at £500/point improvement in Ox‐PAQ routine activities domain score (95% CI)	NMB (£) at £1000/point improvement in Ox‐PAQ routine activities domain score (95% CI)
NHS and PSS perspective									
Usual care	274	1378.58 (1114.89. 1642.27)	52.17 (48.95, 55.38)						
LISTEN intervention	270	1869.57 (1608.18, 2130.96)	49.59 (46.87, 52.31)	490.99 (127.97, 854.00)	−2.536 (−6.50, 1.43)	−210.54 (−771.30, 350.21)	38.42 (−860.36, 937.20)	785.32 (−1278.38, 2849.03)	2030.16 (−2041.77, 6102.09)

*Notes:* Results from the multiply imputed dataset. Analysis models are adjusted for site, perspective, total baseline costs and baseline Ox‐PAQ routine activities domain score.

Abbreviations: CI, confidence interval; NHS, National Health Service; PSS, personal social services.

### 3.4 Scenario Analysis

The scenario analysis, presented in Table 6, considered the intervention and implementation cost of the LISTEN intervention and the resources required for patients living with long Covid in a routine health and care setting.

**Table 6:** Scenario analysis—LISTEN intervention in a routine health and care setting.

Allocation arm	*n*	Mean adjusted cost (£) (95% CI)	Mean adjusted QALY (95% CI)	Incremental cost (£) (95% CI)	Incremental QALY (95% CI)	NMB (£) at £20,000/QALY (95% CI)	NMB (£) at £30,000/QALY (95% CI)
NHS and PSS perspective							
Usual care	274	1399.74 (1131.51, 1667.97)	0.126 (0.120, 0.132)				
Intervention	270	1495.88 (1240.34, 1751.42)	0.131 (0.126, 0.137)	130.29 (−233.62, 494.19)	0.005 (−0.004, 0.014)	2.90 (−419.65, 425.44)	58.25 (−409.39, 525.88)
Societal costs							
Usual care	274	7032.38 (6163.31, 7901.44)	0.126 (0.120, 0.132)				
Intervention	270	6141.85 (5422.42, 6861.28)	0.131 (0.126, 0.137)	−890.52 (−2013.30, 232.25)	0.005 (−0.004, 0.014)	1438.98 (205.61, 2672.33)	1494.32 (236.17, 2752.47)

Abbreviations: CI, confidence interval; NHS, National Health Service; PSS, personal social services; QALY, quality‐adjusted life year.

Practitioner training costs were allocated over a wider population, assuming that each practitioner could deliver the self‐management support programme to 24 patients over a 1‐year period. It was assumed that practitioners experienced in delivering the LISTEN intervention would require less time for both session preparation and session notes writing, reduced from 30 min each to 15 min each. No change to the session delivery time or the cost of the LISTEN intervention book was considered. Table 7 shows total implementation costs in a routine setting for the LISTEN intervention.

**Table 7:** Implementation costs for routine setting scenario analysis.

Cost component	Total units used	Total cost
Total cost of practitioner training and support	n/a	£64,460.71
Cost per practitioner trained	70 practitioners	£920.87
Cost per patient (assuming 24 patients per year)	24 patients	£38.37
Intervention delivery		
Session preparation (15 min assumed)	628.00 h	£17,221.45
Session delivery	1256 sessions	£64,263.28
Session notes writing (15 min assumed)	628.00 h	£17,221.45
Total cost of intervention delivery	n/a	£98,706.17
Cost per session	1256 sessions	£78.59
Cost per patient (based on mean 5.3 sessions)	5.3 sessions	£416.48
Cost per LISTEN book (including postage of £5)	1 book	£11.09
Total cost per patient	1 patient	£465.94

The routine setting implementation cost reduces total costs for the intervention arm and was assumed to have no impact on QALYs or other healthcare resource use compared to what was observed in the trial. For the NHS/PSS perspective, the LISTEN intervention continues to have higher total mean costs and QALYs compared to usual care resulting in an ICER of £26,058 per QALY gained and incremental NMBs of £3 (95% CI, −£420 to £425) and £58 (95% CI, −£409 to £526) at the £20,000 and £30,000 willingness‐to‐pay thresholds, respectively. The LISTEN intervention remained dominant from the societal perspective.

## 4 Discussion

Our within‐trial CEA of the LISTEN self‐management intervention for people living with long Covid revealed that, whilst the intervention group accumulated more QALYs than those randomised to usual care, the LISTEN intervention was more costly from an NHS and PSS perspective and thus not cost‐effective based on accepted UK thresholds over the 3‐month trial follow‐up period. The difference in QALYs gained between arms was small, with participants in the LISTEN arm gaining an average equivalent to 10.9 additional hours of full health over 3 months. The clinical analysis similarly found limited evidence of a clinically meaningful benefit [19].

From a societal perspective, accounting for patient‐incurred costs as well as productivity losses and informal care, the LISTEN Intervention was found to dominate usual care, being less costly and more effective. This difference in results between NHS/PSS and societal perspectives highlights the considerable impact that long Covid had on participants' ability to engage in work and normal activities. A corresponding reduction in private healthcare costs was also observed for the LISTEN intervention group, suggesting that providing structured support to people with long Covid reduces the financial burden the condition imposes on individuals. This shows that, despite methodological challenges, taking a broader societal perspective to the economic analysis of rehabilitation interventions such as LISTEN provides context beyond health needed to inform decision‐making. Nevertheless, the between‐group differences at 6 weeks and 3 months were not statistically significant with wide CIs, partly due to a few individuals reporting very high costs.

### 4.1 Impact

A large difference in cost‐effectiveness probabilities was observed between the NHS/PSS and societal perspectives. This is primarily related to the small difference in QALYs, combined with small changes in the cost difference. The 3‐month time horizon in this analysis is likely insufficient to have realised the full extent of the differences in either costs or benefits of personalised self‐management support. Long Covid appears to have similar traits to other long‐term conditions, such as myalgic encephalomyelitis/chronic fatigue syndrome [36, 37], with a persistent impact on quality of life and ongoing demands on health and care resources. Extrapolations of the costs and benefits beyond the short follow‐up period of 3 months may further improve the relative cost‐effectiveness of the LISTEN intervention. A longer‐term follow‐up study would help understand the mechanisms influencing changes in quality of life, the demands on the health and care sector, and the wider impact on society.

The largest proportion of total costs was attributable to work, informal care and normal activities. The detrimental impact of long Covid on productivity and informal care has previously been documented [9, 38], with approximately 80,000 people estimated to have left the UK workforce due to long Covid [5]. Participants allocated to the LISTEN intervention lost fewer work hours, received fewer hours of informal care, and lost fewer hours of normal activities. The reduction in lost work hours for the LISTEN group is associated with small utility gains. Therefore, as participants continue to manage long Covid symptoms, some trade‐off may exist between increased work and better utility scores. Additionally, the LISTEN intervention reduced the need for participants to seek additional care and increased their ability to resume usual activities, which reduces the financial burden on people with long Covid, as well as lost productivity to society. Most informal care is provided by partners and spouses [38]. Reducing the burden on informal carers not only reduces the societal costs associated with long Covid but generates additional benefits in increased work, leisure and well‐being for caregivers, which this study does not capture.

The cost‐effectiveness results of the LISTEN intervention are consistent with those published from the REGAIN trial, which found a rehabilitation intervention for adults discharged from the hospital following Covid‐19 to be modestly cost‐effective. Notably, participants involved in the LISTEN trial had not been admitted to the hospital for treatment of Covid‐19 symptoms. Whilst evidence of effective treatments continues to emerge [15, 16, 17, 18], many studies differ in terms of their definition of long Covid, population or inclusion criteria. Alongside the range of symptoms associated with long Covid, no standard‐of‐care has yet been established. Future research may wish to consider targeting interventions to those most at risk of developing long Covid and personalising treatments based on clinical or serological variables [39, 40].

### 4.2 Strengths and Weaknesses

The LISTEN randomised controlled trial started at a time when little was known about long Covid, the availability of services was variable or non‐existent, and there was no consensus on condition‐specific outcome measures for research or clinical use. Many participants would have developed symptoms before the availability of vaccines or specific services for people with long Covid. While some evidence is slowly emerging on the effectiveness of different interventions to help people living with long Covid manage their symptoms and everyday life [15, 16, 17, 18], many studies are still ongoing [41, 42, 43], and cost‐effectiveness evidence is still largely missing.

A recent report suggests that 3% of the UK population currently live with long Covid, with limited access to services and a lack of long‐term solutions [9]. People living with long Covid were found to have around 43% higher primary care costs compared to people without long Covid symptoms [44], and long Covid was associated with substantial increases in health and care resource use and costs [45]. Effective treatment and management for long Covid may therefore have long‐run benefits for the health and care system and the wider economy [9].

Our analysis indicates that a personalised self‐management support intervention for people with long Covid may be highly cost‐effective from a societal perspective. However, the study has some key limitations. Research has indicated that long Covid may further exacerbate health and quality of life inequalities with differential prevalence and increased impact on deprived and minority ethnic populations [38, 46, 47]. As the majority of the study population was white, and 72% were female, it is unclear whether the findings of this study are generalisable to the wider population. Furthermore, participants in the LISTEN study had not previously been admitted to hospital with Covid‐19; therefore, the results are not generalisable to people with long Covid following discharge from hospital. Future studies could consider distributional cost‐effectiveness analyses to consider the impact of health inequalities on the provision of long Covid services.

The adapted CSRI used to collect health and care resource use within the LISTEN study was comprehensive and was designed with support from a range of stakeholders, including public members of the co‐design team living with long Covid. This process resulted in a lengthy CSRI, which was burdensome for some trial participants to complete; included the potential for double counting of resource use items; and likely resulted in increased volumes of missing data. Whilst this is likely to have affected both arms, it impacts the reliability of the resource costs used in the analysis and the magnitude of cost‐effectiveness observed.

Furthermore, the complexity of long Covid, with the uniqueness and variability of symptoms [48], may have caused difficulties for participants to identify which resource use items were directly associated with their condition. This may have resulted in confusion and an inability to distinguish between items on the resource use questionnaire, further impacting the reliability of the costing methodology employed.

The outcomes from the LISTEN trial are limited to data collected at the 3‐month endpoint. Due to the chronic nature of long Covid, the analysis is likely insufficient to provide the full extent of the costs and effects of the intervention, and consideration of the longer term would be beneficial. The REGAIN trial [14], which had a follow‐up period of 12 months, similarly found modest evidence of cost‐effectiveness of a rehabilitation programme for people discharged from hospital after admission for Covid‐19; however, beyond 3 months, differences in costs and effects were small and not significant. The results from both the LISTEN and REGAIN trials could contribute to future meta‐analyses to help inform future analyses of the longer‐term costs and effects of self‐management and rehabilitation interventions for people with long Covid.

### 4.3 Summary

In summary, the results indicate that from a societal perspective, the LISTEN intervention was highly cost‐effective. This finding should, however, be interpreted with caution based on the short follow‐up horizon, small differences in costs and effects, and a lack of cost‐effectiveness evidence from an NHS/PSS perspective. Similarly, the clinical analysis of the LISTEN intervention supported a mechanism of impact but was unable to provide firm conclusions regarding a clinically meaningful benefit [19]. Given that recent estimates suggest long Covid could result in macroeconomic costs of £1.5bn each year in the United Kingdom mainly due to productivity losses [9], any intervention that could reduce health and care costs, as well as improve people's ability to work and undertake their daily activities, should be given serious consideration by decision‐makers to help limit the burden of long Covid on the NHS, people and society. Further research is, however, needed to evaluate rehabilitation interventions for people with long Covid in terms of their longer‐term cost‐effectiveness.

## Author Contributions

**Shaun R. S. Harris:** writing – original draft, writing – review and editing, formal analysis, methodology, validation. **Bernadette Sewell:** conceptualisation, funding acquisition, writing – review and editing, supervision, validation, methodology. **Monica Busse‐Morris:** writing – review and editing, funding acquisition, project administration, conceptualisation, investigation. **Adrian Edwards:** writing – review and editing, funding acquisition, project administration. **Fiona Jones:** writing – review and editing, funding acquisition, project administration, conceptualisation, investigation. **Fiona Leggat:** writing – review and editing, investigation. **Philip Pallman:** validation, investigation, methodology, writing – review and editing. **Deborah Fitzsimmons:** writing – review and editing, supervision, methodology.

## Disclosure

The views expressed are those of the authors and not necessarily those of the NIHR or the Department of Health and Social Care. All researchers can confirm their independence from funders, and all authors, external and internal, had full access to all the data (including statistical reports and tables) in the study and can take responsibility for the integrity of the data and the accuracy of the data analysis.

## Ethics Statement

This study was approved on 13 December 2021 by the Research Ethics Committee (REC) for Wales (Wales REC 7), recognised by the United Kingdom Ethics Committee Authority (UKECA), REC reference: 21/WA/0368.

## Conflicts of Interest

All authors have completed the ICMJE uniform disclosure form at www.icmje.org/coi_disclosure.pdf and declare no support from any commercial organisation for the submitted work; no financial relationships with any organisations that might have an interest in the submitted work in the previous 3 years; and no other relationships or activities that could appear to have influenced the submitted work.

Authors are chief investigators or co‐investigators on various current research grants from the UK National Institute for Health and Care Research. The Centre for Trials Research receives infrastructure funding from Health and Care Research Wales. PRIME Centre Wales and the former Wales Covid‐19 Evidence Centre (now Health & Care Research Wales Evidence Centre) receive infrastructure funding from Health and Care Research Wales.

Up until March 2024, F.J.'s research was supported by the NIHR Applied Research Collaboration South London at King's College Hospital NHS Foundation Trust.

M.B. was a member of the NIHR Health Technology Assessment Commissioned Funding Committee from 2020 to 2023 and is a current member of the NIHR Advanced Fellowship panel and MRC Clinical Fellowship Panel.

P.P. is a current member of the MRC and NIHR Efficacy and Mechanism Evaluation Funding Committee.

F.J. is the founder and CEO of Bridges Self‐Management, a non‐profit social enterprise that was involved in the co‐design of the LISTEN intervention and the training of the LISTEN intervention.

## Supporting information

Supplementary Material **Table 2a:** Hospital Services – Mean Resource Use. **Table 2b:** GP and Practice Nurse Resource Use. **Table 2c:** Health and care resource use – Other Community Health Services. **Table 2d:** Health and care resource use – Personal and Social Services. **Table 2e:** Health and care resource use – Mental Health Services. **Table 2f:** Health and care resource use – Work, Informal Care, and Normal Activities.

Supplementary Material

## Data Availability

Data are available upon reasonable request from: ctrdatasamplerequests@cardiff.ac.uk. CTR is a signatory of AllTrials and aims to make its research data available wherever possible. Data requests undergo a review process to ensure that the proposal complies with patient confidentiality, regulatory and ethical approvals and any terms and conditions associated with the data.
