# Supplementary material for: Cost‐Effectiveness of a Personalised Self‐Management Intervention for People Living With Long Covid: The LISTEN Randomised Controlled Trial
Source: Health Expect. 2025 Aug 4;28(4):e70357. doi: 10.1111/hex.70357 (PMC12818045; doi:10.1111/hex.70357)
Supplement: Supplementary file 1 — Supplementary Material Table 2a: Hospital Services – Mean Resource Use. Table 2b: GP and Practice Nurse Resource Use. Table 2c: Health and care resource use – Other Community Health Services. Table 2d: Health and care resource use – Personal and Social Services. Table 2e: Health and care resource use – Mental Health Services. Table 2f: Health and care resource use – Work, Informal Care, and Normal Activities. [file HEX-28-e70357-s002.docx]

Table 2: Mean resource use by category and randomised allocation

Table 2a: Hospital Services – Mean Resource Use

|  | **LISTEN Intervention**  **N=270** | | | **Usual Care**  **N=274** | | |
| --- | --- | --- | --- | --- | --- | --- |
|  | **Time** | | | **Time** | | |
|  | **3-months up to baseline** | **Baseline to 6-weeks** | **6-weeks to 3-months** | **3-months up to baseline** | **Baseline to 6-weeks** | **6-weeks to 3-months** |
|  | **Mean Resource Use**  **(95% CI)**  **[n]** | **Mean Resource Use**  **(95% CI)**  **[n]** | **Mean Resource Use**  **(95% CI)**  **[n]** | **Mean Resource Use**  **(95% CI)**  **[n]** | **Mean Resource Use**  **(95% CI)**  **[n]** | **Mean Resource Use**  **(95% CI)**  **[n]** |
| Long Covid Clinic (number of visits) | 0.38  (0.31, 0.46)  [267] | 0.07  (0.04, 0.11)  [208] | 0.12  (0.07, 0.18)  [196] | 0.38  (0.31, 0.46)  [272] | 0.23  (0.17, 0.31)  [222] | 0.29  (0.22, 0.38)  [183] |
| Outpatient (number of visits) | 0.88  (0.77, 1.00)  [259] | 0.37  (0.30, 0.47)  [209] | 0.40  (0.31, 0.50)  [181] | 0.76  (0.66, 0.87)  [270] | 0.36  (0.28, 0.44)  [222] | 0.54  (0.43, 0.66)  [166] |
| Inpatient (number of stays) | 0.00  (0.00, 0.02)  [264] | 0.00  (0.00, 0.03)  [210] | 0.01  (0.00, 0.03)  [191] | 0.00  (0.00, 0.02)  [269] | 0.00  (0.00, 0.03)  [221] | 0.02  (0.01, 0.06)  [182] |
| Day cases (number of procedures) | 0.04  (0.02, 0.07)  [264] | 0.00  (0.00, 0.03)  [209] | 0.04  (0.02, 0.08)  [181] | 0.04  (0.02, 0.07)  [271] | 0.03  (0.01, 0.06)  [222] | 0.02  (0.00, 0.05)  [167] |
| Emergency department (number of visits) | 0.09  (0.06, 0.13)  [265] | 0.01  (0.00, 0.04)  [210] | 0.02  (0.01, 0.05)  [188] | 0.08  (0.05, 0.12)  [272] | 0.03  (0.01, 0.07)  [221] | 0.05  (0.02, 0.09)  [171] |
| Hospital doctor phone calls (number of calls) | 0.08  (0.05, 0.12)  [264] | 0.01  (0.00, 0.04)  [209] | 0.09  (0.05, 0.14)  [191] | 0.15  (0.11, 0.20)  [268] | 0.08  (0.05, 0.12)  [220] | 0.09  (0.05, 0.15)  [176] |
| Hospital nurse phone calls (number of calls) | 0.07  (0.04, 0.11)  [264] | 0.01  (0.00, 0.04)  [209] | 0.01  (0.00, 0.04)  [192] | 0.05  (0.03, 0.08)  [267] | 0.04  (0.02, 0.07)  [219] | 0.02  (0.00, 0.05)  [176] |

Table 2b: GP and Practice Nurse Resource Use

|  | **LISTEN Intervention**  **N=270** | | | **Usual Care**  **N=274** | | |
| --- | --- | --- | --- | --- | --- | --- |
|  | **Time** | | | **Time** | | |
|  | **3-months up to baseline** | **Baseline to 6-weeks** | **6-weeks to 3-months** | **3-months up to baseline** | **Baseline to 6-weeks** | **6-weeks to 3-months** |
|  | **Mean Resource Use**  **(95% CI)**  **[n]** | **Mean Resource Use**  **(95% CI)**  **[n]** | **Mean Resource Use**  **(95% CI)**  **[n]** | **Mean Resource Use**  **(95% CI)**  **[n]** | **Mean Resource Use**  **(95% CI)**  **[n]** | **Mean Resource Use**  **(95% CI)**  **[n]** |
| GP appointments at practice (number of appointments) | 0.37  (0.30, 0.45)  [261] | 0.09  (0.05, 0.14)  [207] | 0.09  (0.06, 0.15)  [192] | 0.28  (0.22, 0.35)  [269] | 0.12  (0.08, 0.17)  [218] | 0.15  (0.10, 0.22)  [177] |
| Home visits from GP (number of visits) | 0.00  (0.00, 0.02)  [264] | 0.00  (0.00, 0.00)  [209] | 0.00  (0.00, 0.00)  [192] | 0.00  (0.00, 0.00)  [270] | 0.02  (0.01, 0.05)  [218] | 0.02  (0.00, 0.05)  [176] |
| Telephone/video call with a GP (number of calls) | 0.33  (0.26, 0.41)  [264] | 0.08  (0.05, 0.13)  [208] | 0.06  (0.03, 0.11)  [190] | 0.35  (0.28, 0.43)  [268] | 0.12  (0.08, 0.18)  [217] | 0.13  (0.08, 0.19)  [176] |
| Appointment with nurse at the GP practice (number of visits) | 0.14  (0.10, 0.19)  [263] | 0.07  (0.04, 0.11)  [207] | 0.03  (0.01, 0.07)  [191] | 0.13  (0.09, 0.18)  [270] | 0.06  (0.03, 0.10)  [218] | 0.03  (0.01, 0.07)  [175] |
| Telephone/video call with a GP practice nurse (number of calls) | 0.05  (0.03, 0.09)  [263] | 0.00  (0.00, 0.00)  [209] | 0.01  (0.00, 0.03)  [191] | 0.03  (0.01, 0.06)  [270] | 0.00  (0.00, 0.00)  [214] | 0.02  (0.00, 0.05)  [177] |
| Home visit from a district or community nurse (number of visits) | 0.00  (0.00, 0.00)  [264] | 0.00  (0.00, 0.03)  [209] | 0.00  (0.00, 0.00)  [192] | 0.00  (0.00, 0.00)  [270] | 0.02  (0.01, 0.05)  [220] | 0.01  (0.00, 0.03)  [177] |
| Telephone calls to NHS 111 or NHS Direct Wales (number of calls) | 0.02  (0.01, 0.05)  [264] | 0.00  (0.00, 0.00)  [209] | 0.00  (0.00, 0.00)  [192] | 0.01  (0.00, 0.03)  [271] | 0.00  (0.00, 0.00)  [220] | 0.02  (0.01, 0.06)  [177] |

Table 2c: Health and care resource use – Other Community Health Services

|  | **LISTEN Intervention**  **N=270** | | | **Usual Care**  **N=274** | | |
| --- | --- | --- | --- | --- | --- | --- |
|  | **Time** | | | **Time** | | |
|  | **3-months up to baseline** | **Baseline to 6-weeks** | **6-weeks to 3-months** | **3-months up to baseline** | **Baseline to 6-weeks** | **6-weeks to 3-months** |
|  | **Mean Resource Use**  **(95% CI)**  **[n]** | **Mean Resource Use**  **(95% CI)**  **[n]** | **Mean Resource Use**  **(95% CI)**  **[n]** | **Mean Resource Use**  **(95% CI)**  **[n]** | **Mean Resource Use**  **(95% CI)**  **[n]** | **Mean Resource Use**  **(95% CI)**  **[n]** |
| Attendance at home by paramedic (999 call) not requiring transfer to emergency (number of visits) | 0.00  (0.00, 0.00)  [265] | 0.00  (0.00, 0.00)  [209] | 0.00  (0.00, 0.00)  [192] | 0.00  (0.00, 0.00)  [270] | 0.00  (0.00, 0.00)  [220] | 0.00  (0.00, 0.00)  [177] |
| Community pharmacist (number of visits) | 0.07  (0.04, 0.11)  [264] | 0.03  (0.01, 0.06)  [210] | 0.02  (0.00, 0.05)  [192] | 0.12  (0.08, 0.17)  [271] | 0.07  (0.04, 0.11)  [220] | 0.01  (0.00, 0.03)  [177] |
| Appointment with an NHS physiotherapist at a health centre/GP practice (number of visits) | 0.09  (0.06, 0.14)  [263] | 0.00  (0.00, 0.00)  [206] | 0.04  (0.01, 0.08)  [192] | 0.07  (0.05, 0.11)  [271] | 0.00  (0.00, 0.00)  [218] | 0.09  (0.05, 0.15)  [177] |
| Appointment with an NHS physiotherapist at your own home (number of contacts) | 0.05  (0.02, 0.08)  [265] | 0.01  (0.00, 0.03)  [208] | 0.02  (0.01, 0.05)  [192] | 0.02  (0.01, 0.04)  [270] | 0.00  (0.00, 0.00)  [220] | 0.01  (0.00, 0.03)  [177] |
| Remote consultation with an NHS physiotherapist via computer or phone (number of contacts) | 0.08  (0.05, 0.12)  [265] | 0.00  (0.00, 0.00)  [206] | 0.02  (0.00, 0.05)  [181] | 0.01  (0.00, 0.04)  [271] | 0.00  (0.00, 0.00)  [217] | 0.04  (0.01, 0.08)  [164] |
| Appointment with a private physiotherapist (number of contacts) | 0.02  (0.01, 0.04)  [265] | 0.02  (0.01, 0.06)  [210] | 0.01  (0.00, 0.04)  [192] | 0.06  (0.03, 0.09)  [271] | 0.13  (0.09, 0.19)  [220] | 0.05  (0.02, 0.09)  [176] |
| Appointment with an NHS occupational therapist at a health centre/GP practice (number of visits) | 0.04  (0.02, 0.07)  [265] | 0.00  (0.00, 0.00)  [209] | 0.01  (0.00, 0.03)  [192] | 0.07  (0.05, 0.11)  [271] | 0.04  (0.02, 0.07)  [220] | 0.00  (0.00, 0.00)  [177] |
| Appointment with an NHS occupational therapist at your own home (number of contacts) | 0.03  (0.01, 0.06)  [265] | 0.00  (0.00, 0.00)  [207] | 0.01  (0.00, 0.03)  [192] | 0.02  (0.01, 0.04)  [270] | 0.00  (0.00, 0.00)  [219] | 0.00  (0.00, 0.00)  [176] |
| Remote consultation with an NHS occupational therapist via computer or phone (number of contacts) | 0.05  (0.02, 0.08)  [265] | 0.04  (0.02, 0.08)  [210] | 0.02  (0.01, 0.05)  [192] | 0.06  (0.03, 0.10)  [271] | 0.02  (0.00, 0.05)  [220] | 0.03  (0.01, 0.07)  [177] |
| Appointment with a private occupational therapist (number of contacts) | 0.02  (0.01, 0.04)  [263] | 0.00  (0.00, 0.00)  [210] | 0.01  (0.00, 0.04)  [192] | 0.03  (0.01, 0.05)  [271] | 0.00  (0.00, 0.03)  [220] | 0.00  (0.00, 0.00)  [177] |
| Appointment with an NHS psychologist (number of contacts) | 0.07  (0.04, 0.11)  [265] | 0.00  (0.00, 0.03)  [210] | 0.05  (0.02, 0.10)  [192] | 0.08  (0.05, 0.12)  [271] | 0.02  (0.01, 0.05)  [219] | 0.02  (0.01, 0.06)  [176] |
| Other community-based care (number of contacts) | 0.00  (0.00, 0.00)  [254] | 0.00  (0.00, 0.00)  [207] | 0.03  (0.01, 0.07)  [188] | 0.07  (0.04, 0.10)  [261] | 0.00  (0.00, 0.00)  [210] | 0.00  (0.00, 0.00)  [171] |
| Long COVID rehabilitation programme (number of sessions) | 1.08  (0.96, 1.22)  [265] | 0.44  (0.36, 0.54)  [210] | 0.30  (0.23, 0.39)  [195] | 1.14  (1.02, 1.28)  [270] | 1.00  (0.87, 1.14)  [222] | 0.97  (0.83, 1.13)  [182] |
| Private, alternative or complementary health services (number of visits/contacts) | 1.83  (1.67, 2.00)  [260] | 0.53  (0.43, 0.64)  [210] | 0.53  (0.43, 0.64)  [194] | 2.67  (2.48, 2.87)  [270] | 1.76  (1.59, 1.95)  [218] | 1.09  (0.95, 1.26)  [179] |
| Medications (number of prescribed medications) | 1.01  (0.85, 1.17)  [263] | 0.73  (0.58, 0.88)  [205] | 0.78  (0.56, 1.00)  [119] | 0.98  (0.82, 1.13)  [264] | 0.59  (0.45, 0.73)  [212] | 0.66  (0.51, 0.82)  [176] |

Table 2d: Health and care resource use – Personal and Social Services

|  | **LISTEN Intervention**  **N=270** | | | **Usual Care**  **N=274** | | |
| --- | --- | --- | --- | --- | --- | --- |
|  | **Time** | | | **Time** | | |
|  | **3-months up to baseline** | **Baseline to 6-weeks** | **6-weeks to 3-months** | **3-months up to baseline** | **Baseline to 6-weeks** | **6-weeks to 3-months** |
|  | **Mean Resource Use**  **(95% CI)**  **[n]** | **Mean Resource Use**  **(95% CI)**  **[n]** | **Mean Resource Use**  **(95% CI)**  **[n]** | **Mean Resource Use**  **(95% CI)**  **[n]** | **Mean Resource Use**  **(95% CI)**  **[n]** | **Mean Resource Use**  **(95% CI)**  **[n]** |
| Appointment with a social worker at a centre/GP practice (number of contacts) | 0.00  (0.00, 0.00)  [265] | 0.00  (0.00, 0.00)  [210] | 0.00  (0.00, 0.00)  [192] | 0.00  (0.00, 0.00)  [271] | 0.00  (0.00, 0.00)  [220] | 0.03  (0.01, 0.07)  [177] |
| Home visit with a social worker (number of contacts) | 0.01  (0.00, 0.03)  [265] | 0.00  (0.00, 0.03)  [209] | 0.00  (0.00, 0.00)  [192] | 0.00  (0.00, 0.00)  [271] | 0.01  (0.00, 0.03)  [221] | 0.00  (0.00, 0.00)  [177] |
| Telephone/video call with a social worker (number of calls) | 0.01  (0.00, 0.03)  [265] | 0.00  (0.00, 0.00)  [210] | 0.00  (0.00, 0.00)  [192] | 0.00  (0.00, 0.00)  [270] | 0.00  (0.00, 0.00)  [220] | 0.01  (0.00, 0.03)  [177] |
| Home care worker visits (minutes per week) | 0.00  (0.00, 0.00)  [264] | 0.00  (0.00, 0.00)  [210] | 0.16  (0.11, 0.23)  [184] | 0.17  (0.12, 0.22)  [272] | 0.13  (0.09, 0.19)  [223] | 0.00  (0.00, 0.00)  [210] |

Table 2e: Health and care resource use – Mental Health Services

|  | **LISTEN Intervention**  **N=270** | | | **Usual Care**  **N=274** | | |
| --- | --- | --- | --- | --- | --- | --- |
|  | **Time** | | | **Time** | | |
|  | **3-months up to baseline** | **Baseline to 6-weeks** | **6-weeks to 3-months** | **3-months up to baseline** | **Baseline to 6-weeks** | **6-weeks to 3-months** |
|  | **Mean Resource Use**  **(95% CI)**  **[n]** | **Mean Resource Use**  **(95% CI)**  **[n]** | **Mean Resource Use**  **(95% CI)**  **[n]** | **Mean Resource Use**  **(95% CI)**  **[n]** | **Mean Resource Use**  **(95% CI)**  **[n]** | **Mean Resource Use**  **(95% CI)**  **[n]** |
| Mental health counsellor (number of contacts) | 0.30  (0.24, 0.38)  [266] | 0.10  (0.06, 0.15)  [210] | 0.07  (0.04, 0.12)  [192] | 0.33  (0.26, 0.40)  [268] | 0.15  (0.11, 0.22)  [220] | 0.21  (0.15, 0.29)  [183] |
| Mental health community psychiatrist (number of contacts) | 0.02  (0.01, 0.04)  [265] | 0.00  (0.00, 0.03)  [209] | 0.00  (0.00, 0.00)  [192] | 0.03  (0.01, 0.05)  [268] | 0.01  (0.00, 0.04)  [220] | 0.03  (0.01, 0.07)  [182] |
| Mental health psychologist (number of contacts) | 0.09  (0.06, 0.14)  [265] | 0.01  (0.00, 0.03)  [210] | 0.01  (0.00, 0.04)  [192] | 0.07  (0.05, 0.12)  [267] | 0.07  (0.04, 0.11)  [219] | 0.05  (0.03, 0.10)  [182] |
| Mental health community psychiatric nurse (number of contacts) | 0.00  (0.00, 0.02)  [266] | 0.00  (0.00, 0.03)  [210] | 0.00  (0.00, 0.00)  [192] | 0.01  (0.00, 0.03)  [267] | 0.01  (0.00, 0.03)  [220] | 0.04  (0.02, 0.09)  [182] |
| Community mental health team member (number of contacts) | 0.09  (0.06, 0.14)  [265] | 0.00  (0.00, 0.00)  [205] | 0.07  (0.04, 0.12)  [192] | 0.05  (0.03, 0.09)  [268] | 0.00  (0.00, 0.00)  [213] | 0.03  (0.01, 0.06)  [182] |
| NHS mental health helpline (number of calls) | 0.02  (0.01, 0.04)  [266] | 0.02  (0.01, 0.06)  [210] | 0.01  (0.00, 0.03)  [192] | 0.01  (0.00, 0.03)  [268] | 0.02  (0.00, 0.05)  [220] | 0.03  (0.01, 0.07)  [182] |
| Mental health memory services (number of contacts) | 0.01  (0.00, 0.03)  [266] | 0.00  (0.00, 0.00)  [210] | 0.00  (0.00, 0.00)  [192] | 0.00  (0.00, 0.00)  [268] | 0.00  (0.00, 0.00)  [219] | 0.00  (0.00, 0.00)  [182] |
| Other mental health service (number of contacts) | 0.00  (0.00, 0.00)  [261] | 0.01  (0.00, 0.04)  [203] | 0.00  (0.00, 0.00)  [190] | 0.00  (0.00, 0.00)  [264] | 0.00  (0.00, 0.00)  [217] | 0.00  (0.00, 0.00)  [180] |
| Private mental health contacts (number of contacts) | 0.34  (0.27, 0.42)  [263] | 0.12  (0.08, 0.17)  [211] | 0.10  (0.06, 0.15)  [193] | 0.43  (0.36, 0.52)  [269] | 0.18  (0.13, 0.25)  [219] | 0.36  (0.27, 0.46)  [177] |

Table 2f: Health and care resource use – Work, Informal Care, and Normal Activities

|  | **LISTEN Intervention**  **N=270** | | | **Usual Care**  **N=274** | | |
| --- | --- | --- | --- | --- | --- | --- |
|  | **Time** | | | **Time** | | |
|  | **3-months up to baseline** | **Baseline to 6-weeks** | **6-weeks to 3-months** | **3-months up to baseline** | **Baseline to 6-weeks** | **6-weeks to 3-months** |
|  | **Mean Resource Use**  **(95% CI)**  **[n]** | **Mean Resource Use**  **(95% CI)**  **[n]** | **Mean Resource Use**  **(95% CI)**  **[n]** | **Mean Resource Use**  **(95% CI)**  **[n]** | **Mean Resource Use**  **(95% CI)**  **[n]** | **Mean Resource Use**  **(95% CI)**  **[n]** |
| Lost work hours (total hours) | 72.67  (71.57, 73.77)  [233] | 31.53  (30.74, 32.33)  [193] | 21.36  (20.68, 22.06)  [173] | 85.29  (84.15, 86.44)  [251] | 30.38  (29.61, 31.16)  [195] | 32.57  (31.69, 33.47)  [160] |
| Hours of care from family and/or friends (total hours) | 67.13  (66.15, 68.13)  [262] | 27.62  (26.91, 28.35)  [207] | 40.47  (39.57, 41.39)  [188] | 62.33  (61.39, 63.28)  [268] | 34.54  (33.76, 35.34)  [214] | 40.47  (39.55, 41.42)  [179] |
| Hours lost from normal activities (total hours) | 160.89  (135.58, 186.19)  [266] | 81.53  (68.18, 94.88)  [209] | 80.46  (65.44, 95.49)  [194] | 179.79  (152.74, 206.85)  [271] | 98.01  (80.84, 115.18)  [215] | 102.63  (82.80, 122.45)  [183] |

Table 2g: Health and care resource use – Private healthcare and patient incurred costs

|  | **LISTEN Intervention**  **N=270** | | | **Usual Care**  **N=274** | | |
| --- | --- | --- | --- | --- | --- | --- |
|  | **Time** | | | **Time** | | |
|  | **3-months up to baseline** | **Baseline to 6-weeks** | **6-weeks to 3-months** | **3-months up to baseline** | **Baseline to 6-weeks** | **6-weeks to 3-months** |
|  | **Total costs (£)**  **(95% CI)**  **[n]** | **Total costs (£)**  **(95% CI)**  **[n]** | **Total costs (£)**  **(95% CI)**  **[n]** | **Total costs (£)**  **(95% CI)**  **[n]** | **Total costs (£)**  **(95% CI)**  **[n]** | **Total costs (£)**  **(95% CI)**  **[n]** |
| Over the counter medication costs | 78.19  (59.49, 96.89)  [187] | 43.59  (30.52, 56.67)  [134] | 34.96  (25.37, 44.55)  [117] | 76.32  (62.05, 90.58)  [204] | 47.32  (35.66, 58.97)  [134] | 63.32  (36.91, 89.73)  [126] |
| Prescription and pre-payment certificate costs | 5.01  (3.83, 6.19)  [262] | 4.08  (2.92, 5.24)  [205] | 4.02  (2.36, 5.68)  [119] | 3.78  (2.77, 4.79)  [264] | 3.26  (2.18, 4.34)  [212] | 3.37  (2.20, 4.54)  [176] |
| Private home care worker cost | 0.00  (0.00, 0.00)  [264] | 0.00  (0.00, 0.00)  [210] | 0.00  (0.00, 0.00)  [194] | 0.11  (-0.11, 0.33)  [272] | 0.62  (-0.60, 1.84)  [223] | 0.57  (-0.56, 1.70)  [184] |
| Additional childcare costs | 11.32  (-1.11, 23.75  [265] | 5.38  (-0.77, 11.52)  [210] | 6.30  (-0.07, 12.67)  [196] | 9.87  (1.74, 18.00)  [271] | 4.32  (0.53, 8.10)  [219] | 3.91  (0.47, 7.35)  [182] |
| Additional mobile or home phone costs | 0.29  (-0.04, 0.63)  [257] | 0.18  (0.01, 0.35)  [186] | 0.08  (-0.08, 0.25)  [178] | 2.03  (0.02, 4.04)  [261] | 0.26  (-0.07, 0.60)  [198] | 0.16  (-0.03, 0.34)  [160] |
| Private physiotherapy and occupational therapist costs | 2.05  (-0.55, 4.65)  [263] | 1.57  (-0.66, 3.80)  [210] | 1.13  (-0.48, 2.73)  [192] | 4.74  (0.67, 8.81)  [271] | 8.89  (-5.59, 23.38)  [220] | 3.00  (-0.29, 6.29)  [176] |
| Privately incurred tests and investigation costs | 96.48  (-68.19, 261.16)  [252] | 8.26  (-2.54, 19.06)  [210] | 10.16  (-2.83, 23.15)  [182] | 10.71  (2.40, 19.02)  [254] | 22.07  (-3.30, 47.45)  [222] | 19.94  (1.11, 38.77)  [166] |
