# Supplementary material for: Cost‐Effectiveness of a Personalised Self‐Management Intervention for People Living With Long Covid: The LISTEN Randomised Controlled Trial
Source: Health Expect. 2025 Aug 4;28(4):e70357. doi: 10.1111/hex.70357 (PMC12818045; doi:10.1111/hex.70357)
Supplement: Supplementary file 2 — Supplementary Material [file HEX-28-e70357-s001.docx]

| **HEALTH ECONOMIC ANALYSIS PLAN** | | |
| --- | --- | --- |
|  | **Trial Title** |  |
| Long Covid Personalised Self-managemenT support EvaluatioN  (LISTEN Trial) | | |
|  | **Trial Summary** |  |
|  |  |  |
| The aim of the two-arm parallel-group unblinded individually randomised controlled LISTEN trial is to evaluate the effectiveness and cost-effectiveness of a personalised self-management support intervention for non-hospitalised people living with long Covid (the ‘LISTEN intervention’) compared to usual care. The LISTEN intervention is a personalised self-management support intervention that draws on evidence from Bridges Self-management theoretically informed by self-efficacy as the most successful foundation for self-management programmes. Over the recruitment period, 474 participants suffering with long Covid will be randomly assigned to the intervention or usual care and asked to complete questionnaires at baseline, six weeks later, and three months later. | | |
|  |  |  |

| **Trial Details** |  |
| --- | --- |
| ISRCTN: ISRCTN36407216  Study Coordination Centre: Cardiff Centre for Trials Research  Chief Investigator: Fiona Jones and Monica Busse-Morris  Trial Statistician: Philip Pallmann and Muhammad Riaz  Study Health Economist: Bernadette Sewell (lead) and Shaun Harris | |
| **Version 1.2, July 2023** | |

Revision History

| **Revision Date** | **Release** | **Summary of Changes** | **Changes Made by** |
| --- | --- | --- | --- |
| 06/2023 | 1.0 | Initial version draft | BS |
| 07/2023 | 1.1 | Updated version internal review (Dr Shaun Harris) | SH, BS |
| 07/2023 | 1.2 | Updated version after internal QA (Prof. Deb Fitzsimmons) | BS |
|  |  |  |  |
|  |  |  |  |
|  |  |  |  |

Agreement

The following people have reviewed the Health Economics Analysis plan and are in agreement with the content.

| **Name** | **Title** | **Version** | **Date** | **Signature** |
| --- | --- | --- | --- | --- |
| Bernadette Sewell | Health Economist |  |  |  |
| Fiona Jones | Chief Investigator |  |  |  |
| Monica Busse-Morris | Chief Investigator |  |  |  |
| Muhammad Riaz | Statistician |  |  |  |

Acronyms and definition of terms

Any acronyms or abbreviations are consistent with the definition of terms in the SAP. Any additional terms are defined, written in full for the first time (abbreviation in brackets), with the abbreviation used subsequently.

Health Economic Analysis Plan and Authorship

This health economic analysis plan (HEAP) was written by Dr Bernadette Sewell and reviewed by Dr Shaun Harris, with internal review by Professor Deb Fitzsimmons (Director of SCHE). The plan will be finalised prior to data analysis after discussion with the Chief Investigators and statistician, and circulation to the Trial Management Group (TMG). The final version will be signed off by the lead author, health economics lead, statistician, and chief investigators. The analysis will be conducted by Bernadette Sewell and Shaun Harris (study health economists) at the Swansea Centre for Health Economics (SCHE) with final internal Quality Assurance undertaken by another senior health economist from SCHE.

# SUMMARY AND OVERVIEW OF THE LISTEN TRIAL

Some people who are infected with Covid-19 suffer from long Covid signs and symptoms and experience a wide variety of longer-term ongoing problems such as fatigue, pains, and difficulty with day-to-day tasks. This means, they may struggle to return to their former lives. This is then made worse by uncertainty and a lack of understanding by some healthcare professionals.

The LISTEN project develops and evaluates an intervention (i.e., a package of self-management support, co-designed with individuals living with long Covid) to ensure that the interventions can be personalised to the individual needs as appropriate.

The study will recruit individuals who are living with long Covid symptoms and will be randomised to the intervention or control group. The control group will receive the usual care and if requested, the LISTEN team will signpost them to long Covid care pathways in their regions. The intervention group will receive the new resources and up to six coaching sessions from the trained rehabilitation practitioners. The healthcare resource use, expenses and time off work will be recorded to understand the economic impact of long Covid and our intervention on society and individuals.

The LISTEN intervention will be evaluated in terms of impact on participation in routine activities, emotional well-being, social participation, fatigue and self-efficacy. The impact on quality of life (effectiveness) and cost-effectiveness of the intervention compared to the usual care will be examined. The primary outcome time-point is at three months following randomization; however, consent will be obtained for longer-term follow-up (beyond the length of the funded evaluation). An internal pilot will assess site opening and recruitment. Intervention acceptability and feasibility will be measured as part of the embedded mixed-methods process evaluation and enable a detailed analysis of implementation enablers and barriers to adoption and sustainability beyond the project timeline. This work will inform and deliver a national implementation support package (for example training programme for rehabilitation teams, web platform, training manuals etc.) ready for scale-up and implementation by the end of the project.

## Conduct of the Health Economic Analysis

This health economics analysis plan (HEAP) has been written as a supplement to the trial statistical analysis plan (SAP; version #.#, dated ##/##/####) and the study protocol [1]. The HEAP outlines the specific methods and procedures to conduct the health economic analysis for the LISTEN trial. Where required, the HEAP will cross-reference back to the SAP and protocol to ensure methods and processes are consistent and outlined procedures are complied with. The HEAP will be updated in line with any updates of the SAP and a final version of the HEAP will be agreed with the TMG and Trial Steering Committee (TSC) before data lock and prior to commencement of the analysis.

Throughout the study, the health economics team will work closely with the trial manager and statistician to ensure agreed processes are in place for data management and cleaning as specified in the SAP. Throughout the study, quality assurance checks will be undertaken to ensure the integrity of the data and compliance with the SAP and HEAP.

## Commencement of Final Health Economic Analysis

Health economic analyses will **only** commence once the study statistician has confirmed, in writing, to the CI and project lead (health economics) that the data has been locked for analyses. Appropriate data management processes will be put in place by the trial team to allow SCHE access to the dataset including secure data transfer and ensuring SCHE has the necessary standard operating procedures (SOP) for data management and statistical analyses. Where trial data needs to be further analysed for the health economic analysis, a full log/decision trail) of this will be kept (e.g. syntax files, to do files, outputs) and be made available to the trial statistician and CI for checking and approval prior to reporting the final health economics results.

# TRIAL OBJECTIVES

LISTEN is a two-arm parallel-group unblinded individually randomised controlled LISTEN trial aiming to evaluate the effectiveness and cost-effectiveness of a personalised self-management support intervention for non-hospitalised people living with long Covid (the ‘LISTEN intervention’) compared to usual care.

All trial details and procedures are contained in the trial protocol [1].

The trial objectives include:

#### Primary Objective

The primary objective is to evaluate the impact of the LISTEN co-designed personalised self-management support intervention on routine activities as assessed by the routine activities’ domain of the Oxford Participation and Activities Questionnaire (Ox-PAQ).

#### Secondary Objectives

Secondary objectives are [1]:

1. To evaluate the impact of the ‘LISTEN intervention’ on emotional well-being as assessed by the relevant domain sub-scale of the Ox-PAQ
2. To evaluate the impact of the ‘LISTEN intervention’ on social engagement as assessed by the relevant domain sub-scale of the Ox-PAQ
3. To evaluate the impact of the ‘LISTEN intervention’ on health-related quality of life as assessed by the Short Form-12 - Health Survey (SF-12)
4. To evaluate the impact of the ‘LISTEN intervention’ on fatigue as measured by the Fatigue Impact Scale (FIS)
5. To gather information on utility (using the EQ-5D-5L questionnaire) and health and care resource use (using an adapted Client Service Receipt Inventory)
6. To assess the cost-effectiveness of the ‘LISTEN intervention’
7. To explore key anticipated mediators of intervention outcome (namely self-efficacy in the context of Covid-19) using the generalised self-efficacy scale (GSES) with additional context-specific questions
8. To conduct a theory-driven detailed process evaluation within the trial using validated implementation scales to assess intervention acceptability, appropriateness, and feasibility

The health economic evaluation will address secondary objectives v and vi.

## Description of Intervention and Control

The intervention consists of personalised self-management support co-designed with people living with long Covid. The control group will receive usual care. A full description of the intervention and control can be found in the protocol (section 11).

## Jurisdiction and perspective

The trial will be conducted in the United Kingdom (UK) National Health Service (NHS) setting which is a publicly funded health care system. All economic analyses will be conducted from an NHS and Personal Social Services (PSS) perspective as recommended by the National Institute for Health and Care Excellence (NICE) [2]. While we will collect costs outside of the health and care sector (accrued by patients), these will not be included in the health economic evaluation but reported descriptively to give a fuller picture of the true cost of long COVID to society.

# ECONOMIC APPROACH

## Aim and objective of the economic evaluation

The aim of the economic evaluation is to consider the within-trial cost-effectiveness of a package of self-management support for individuals living with long Covid.

The primary objectives of the health economic evaluation address trial objectives v and vi, evaluating the impact of the LISTEN intervention package of self-management support versus usual care for patients with long Covid on patient utility and health and care costs and estimating the within-trial cost-effectiveness at 3-months follow-up.

## Overview of economic analysis

Cost-effectiveness will be assessed using individual level data from the LISTEN study.

The within-trial health economic analysis will be conducted alongside the statistical analysis and will consist of the following:

1. A descriptive summary of resource use and costs associated with the intervention implementation;
2. Analysis of the health and personal social services resource use and costs at three months associated with the intervention compared to control;
3. Analysis of health-related quality of life at three months associated with the intervention compared to control based on EQ-5D-5L responses;
4. Between-group differences in costs and QALYs evaluated using appropriate regression techniques;
5. Cost-utility analysis (CUA) that will calculate incremental cost-effectiveness ratio (ICER) for the LISTEN intervention compared to control to establish the cost per quality-adjusted life-year (QALY) gained;
6. A calculation of net monetary benefit (NMB) and net health benefit (NHB) of the LISTEN intervention compared to usual care;
7. Descriptive analysis of costs borne by patients and wider society as additional outcome;
8. A cost-consequences analysis (CCA) that will present outcomes and costs alongside each other in tabular form.
9. Sensitivity analyses (SA) to assess uncertainty in the results using cost-effectiveness acceptability curves and one-way sensitivity analysis (OWSA).

# DATA TO INFORM HEALTH ECONOMIC ANALYSES

The data collection required for the health economic analyses is detailed in the trial protocol [1] and SAP. A variety of methods are utilised including the use of a specific instrument to collect resource utilisation.

## Statistical software for health economic analysis

Microsoft Excel and Stata version 17 or higher will be used for all health economic analyses.

## Identification of outcomes

The primary economic outcomes are costs (including intervention implementation cost and costs of NHS health and care resource) and quality-adjusted life years (QALYs) derived from utility scores obtained from EuroQol 5D-5L (EQ-5D-5L) responses. The trial questionnaire includes the questionnaires to collect resource use and health related quality of life data.

Secondary outcomes included in the health economic analysis (as part of the CCA and/or SA) are:

- Health related quality of life derived using the Shortform-12 questionnaire
- Emotional well-being
- Social engagement
- Fatigue
- Self-sufficiency

## Measurement of outcomes

Cost and health outcomes will be collected at baseline, and 6-week and 3-month follow-up, using participant self-completed questionnaires. Questionnaires can be completed by participants either online or via post.

## Identification of resources and measurement of costs

Relevant NHS and PSS resources expected to be important include: (1) resources required in the development, training, and delivery of the intervention; (2) primary and secondary health and care use; and (3) costs associated with the wider societal impact of long Covid (which will not be included in the primary analysis but reported separately). Where possible, the most recently published unit costs will be used and sourced from publicly available sources including the Personal and Social Services Research Unit (PSSRU) [4], British National Formulary [5], and NHS reference costs [6], or otherwise obtained from local financial records and/or trial records. When a unit cost is not available for the year of analysis (2022/23 to reflect year of data collection), the latest available unit costs will be used with costs inflated to current prices using the NHS cost inflation index (NHSCII) as published by PSSRU [4].

### Intervention Cost

The resource use and costs associated with the intervention itself will be determined by calculating the intervention resource use from trial notes and through discussions with the trial team (as required) and valued as costs in £ sterling. The main cost components are expected to be intervention development, printing and administration, intervention practitioner training, supervision and support, and staff time delivering the intervention which will be costed using published unit costs and other financial information obtained from study records. We do not anticipate additional costs to the participants as a consequence of the intervention and will therefore not collect out-of-pocket expenses as part of the implementation cost. Any opportunity costs associated with the intervention (e.g. staff where people where redeployed from other tasks) will be quantified (if feasible). Any costs related to research will be excluded as these would not be a component of the costs of delivery in standard practice. The costs associated with the intervention shall be categorised into development, training and delivery, with each component tabulated as illustrated by Table A (see Appendix 2). A total cost of the LISTEN intervention and a cost per participant will be presented.

### Costs Associated with Health Care Resource Utilisation

Health service resource use in primary care, secondary care and the community related to symptoms of long Covid will be established via an adapted client service receipt inventory questionnaires (CSRI) [7] to participants in both arms of the trial at baseline and the 6-weeks and 3-month follow-up points. Questions relate to all health service contacts (e.g. hospital appointments, hospital stays, GP contacts, visiting nurse appointments, etc.), tests or investigations, mental health service contacts, social care, medications and treatment during the trial period with a 6-week and 3-month recall period (see Appendix 1). Resource use will be costed using standard published unit costs and valued in £ sterling.

The resources utilised and associated costs will be summarised and tabulated in order to compare the total cost of the intervention with control (see Table B in Appendix 2). Resource usage costs will also be summarised and tabulated by contact type (e.g. GP and community nursing, social services, investigation) (see Table C, Appendix 2). A descriptive analysis at baseline will be presented whilst differences between treatment arms at the 3-month follow-up will be analysed in accordance with the SAP with 95% confidence intervals reported. Total costs will also be used to inform the calculation of the incremental analyses.

Patient recall has been shown to be a valid method for collecting health service resource use data within this period (i.e. up to 3 months) and, as clinical records are often fragmented, and sometimes unavailable across different parts of the health service, patient-reported data is likely to remain more readily available and less costly to collect for research purposes [8].

### Societal Costs

As an NHS and personal social services perspective will be undertaken, wider societal costs including loss of earnings, participant out-of-pocket expenses and opportunity costs incurred by family members will be excluded from the primary analysis. However, in order to allow a detailed analysis of the true cost of long Covid to individuals, we will report the impact on patient out-of-pocket expenses as well as work productivity and carer costs separately as one of the outcomes included in the CCA.

### Health-related Quality of Life

Participants will complete the EuroQol EQ-5D-5L questionnaire at baseline and at the 6-week and 3-month follow-up points. Patient EQ-5D-5L profiles indicated by the participants on the questionnaire will be mapped to the EQ-5D-3L valuation set using the validated mapping function by van Hout et al. [3], allowing a utility score to be calculated for each patient based on UK population values. QALYs will be calculated using an area under the curve approach.

### Patient and Public Involvement

Patients and members of the public co-designed the LISTEN intervention and the LISTEN CSRI. Patient representatives involved in the study helped to develop and review the Client Service Receipt Inventory (CSRI) questionnaire.

# FRAMEWORK FOR THE HEALTH ECONOMIC ANALYSIS

## Overview

Table 1 summarises the methods used in the economic evaluation which are discussed in more detail in the following sections:

Table 1: Summary of Methods used in Health Economic Analysis

| **Aspect of Methodology** | **Strategy for Base-Case** | **Alternative Strategy for Sensitivity Analysis** |
| --- | --- | --- |
| Data set | All randomised participants, analysed on intention-to-treat (ITT) basis | Per protocol analysis |
| Costs included in analysis | Intervention cost  Health and personal social services costs including primary care, secondary care, mental health care, social care, medications and investigation costs  Patient-related costs excluded | Including patient-related costs (e.g. out-of-pocket expenses, loss of earnings) |
| Patient outcomes | Health related quality of life using EQ-5D-5L data | Health related quality of life using SF-12/SF-6D data |
| Missing data | Multiple imputation (see SAP chapter 6.2.7) | Complete case analysis |
| Adjustment for baseline covariates | Regression used to adjust costs and utility outcomes for differences at baseline | No adjustment for baseline covariates |

## Analysis Population

Commensurate with the SAP (section 6.2), participants will be analysed as randomised on an intention-to-treat (ITT) basis.

## Health Economic Analysis Considerations

Similar analysis methods will be used as those detailed in the SAP. Outcomes will be adjusted for baseline covariates following the statistical analysis (e.g., a priori decided set of covariates and covariates identified by the statistical analyses) and baseline utility using appropriate regression techniques. The difference in means between arms adjusted for these variables will be presented with 95% confidence intervals at three months.

## Analysis of resource use and costs

Mean resource use will be estimated and presented for each arm for each resource use category. Standard deviations (SD) and the number of patients included in each category by arm will also be presented. Appropriate regression techniques (e.g., seemingly unrelated regression) will be used to estimate adjusted mean costs and the difference in adjusted mean costs (and associated 95% confidence intervals) between arms. If costs are not normally distributed, appropriate methods will be applied, e.g., non-parametric bootstrapping will be used to derive appropriate 95% confidence intervals around a point estimate of cost per participant in each of the trial arms.

# Post-Trial Modelling

No decision analytic modelling for longer term cost effectiveness will be undertaken as part of the LISTEN trial.

## Missing Data

Clinical studies are typically unable to collect all the intended data. For health economic analysis, missing items relating to health care usage may undervalue total costs, whilst missing outcome data may be intrinsically linked to effects. Missing data may relate to item non-response, where a questionnaire is partially incomplete, or unit non-response where all the information is missing [9].

Missing data will be handled depending upon the prevalence and likely cause of the missingness. The mechanism of missingness will be assessed. For example, if the data is believed to be missing at random (MAR), then multiple imputation methods may be used.

Should multiple imputation be applied, imputations will be combined following Rubin’s rules [10]. Due to the predefined range of outcome scores, predictive mean matching (PMM) will be used to ensure imputed values are consistent with observed data. The plausibility of the MAR assumption will be explored by comparing observed data in participants with and without the item of interest.

## Cost-Effectiveness

Cost-utility analyses will be used to estimate cost per QALY between intervention and control groups. Incremental costs and effects will also be presented in disaggregated format with 95% confidence intervals for results. This comparative analysis of incremental costs and effects can be summarised in terms of an incremental cost-effectiveness ratio (ICER; table D, Appendix 2).

The ICER can be represented as:

$$ICER=\frac{\Delta C}{\Delta E}=\frac{C_{1}-C_{0}}{E_{1}-E_{0}}$$

Where $C_{1}$ and $E_{1}$ are the costs and effects of the intervention arm, and $C_{0}$ and $E_{0}$ are the cost and effects of the Control arm; with $\Delta C$ and $\Delta E$ the incremental costs and effects associated with the intervention compared to Control. Where simple dominance of the intervention is observed (e.g., the intervention is less costly and more effective than Control) ICERs will not be presented.

The reporting of results in the final report will show sufficient information to reproduce the calculations of the ICER.

Furthermore, a cost-consequences analysis will visually compare outcomes and costs of intervention and control in tabular form. An example of the output of the cost-consequences analysis can be found in table E, Appendix 2.

Outputs will also be used to estimate the incremental net monetary benefit (INMB) and net health benefit (INHB) statistic at the standard willingness to pay thresholds as suggested by NICE of both £20,000 and £30,000 per QALY.

## Sampling Uncertainty

Uncertainty will be explored using probabilistic sensitivity analysis and cost-effectiveness acceptability curves to estimate the probability that the intervention is cost-effective at a range of plausible cost-effectiveness thresholds.

## Sensitivity Analyses

Sensitivity analyses will be undertaken to account for the uncertainty in the parameters used in the cost-effectiveness analyses. This will involve making plausible changes to key methodological assumptions in order to understand how changes impact the cost-effectiveness result. Deterministic one-way sensitivity analyses (OWSAs) will be undertaken to examine the impact of changes in key parameters on ICERs by modifying the value of one parameter at a time within a plausible range. Results will be tabulated (Table F, Appendix 2), for comparison against the base-case analysis. Examples include:

- Using per protocol and complete case analysis instead of ITT.
- Inclusion/exclusion of intervention development, training, and ongoing support costs.
- Identifying plausible alternative parameter values for key unit costs.
- QALYs derived from SF-12 questionnaire responses.
- If applicable, different approaches to the handling of missing data.

## Outliers

Cost data relating to healthcare is often highly skewed. The distribution of costs shall be examined to determine the appropriateness of normality assumptions for subsequent statistical analysis. Where appropriate, the impact of removing extreme outliers from the analysis will be considered.

## Discounting

As the follow-up period of the trial does not exceed 12-months, no discounting will be applied for the in-trial analysis of either costs or outcomes.

## Sub-Group Analysis

No sub-group analyses are planned.

# REPORTING/PUBLISHING

## Reporting Standards

The health economics team will work closely with the trial data manager and study statistician throughout the analysis period. The health economic analysis will be undertaken and results checked by the health economic project lead prior to submitting to the TMG for scrutiny and discussion. Where necessary, modifications to the analysis will be conducted following interim results presentation prior to final analyses.

The Consolidated Health Economic Evaluation Reporting Standards (CHEERS) [11] guidelines will be followed when reporting the health economic evaluation.

## Reporting deviations from the HEAP

Any deviations from the HEAP will be documented and justified in the final published report.

# REFERENCES

- - - 1. Potter, C., Leggat, F., Lowe, R. *et al.* (2023). Effectiveness and cost-effectiveness of a personalised self-management intervention for living with long COVID: protocol for the LISTEN randomised controlled trial. *Trials* 24, 75. <https://doi.org/10.1186/s13063-023-07090-w>
      2. National Institute for Health and Care Excellence (2013). Guide to the Methods of Technology Appraisal. Available at: <https://www.nice.org.uk/process/pmg9/chapter/foreword>.
      3. van Hout B, Janssen MF, Feng YS, Kohlmann T, Busschbach J, Golicki D, et al. (2012) Interim scoring for the EQ-5D-5L: mapping the EQ-5D-5L to EQ-5D-3L value sets. Value in health. The Journal of the International Society for Pharmacoeconomics and Outcomes Research, 15(5): 708-15.
      4. Jones, K.C., Weatherly, H., Birch, S., Castelli, A., Chalkley, M., Dargan, A., Forder, J.E., Gao, J., Hinde, S., Markham, S. et al. (2023). Unit Costs of Health and Social Care 2022 Manual. Technical report. Personal Social Services Research Unit (University of Kent) & Centre for Health Economics (University of York), Kent, UK 10.22024/UniKent/01.02.100519.
      5. National Institute for Health and Care Excellence (2023). British National Formulary. Available at: <https://bnf.nice.org.uk/>
      6. NHS England (2022). “NHS Reference Costs 2021/21. Available at: <https://www.england.nhs.uk/costing-in-the-nhs/national-cost-collection/>
      7. Beecham J, Knapp M (1990). Costing mental health services. Psychological Medicine, 20(4), 893-908.
      8. Carpenter, J. R., and Kenward, M. G. (2013) Multiple Imputation and its Application, Chichester, UK: John Wiley & Sons.
      9. White, I. R., and Thompson, S. G. (2005) Adjusting for partially missing basline measurements in randomized trials. *Stat Med,* 24(7): 993-1007.
      10. D. B. Rubin (1987) Multiple Imputation for Nonresponse in Surveys, New Jersey: John Wiley & Sons.
      11. Husereau D, Drummond M, Augustovski F, de Bekker-Grob E, Briggs A H, Carswell C et al. (2022) Consolidated Health Economic Evaluation Reporting Standards 2022 (CHEERS 2022) statement: updated reporting guidance for health economic evaluations. *BMJ,*  376 :e067975.

# APPENDIX 1: Client Service Receipt Inventory (CSRI) as adapted for LISTEN trial and study population

**Use of health and social care services**

In this section we will be asking you some questions about the services you have used and anything you have had to buy **because of your long COVID**. Please **only** include events relating to your **long COVID treatment and care**. If you are not exactly sure, please give your best guess.

**Part 1: USE OF HEALTH AND SOCIAL CARE SERVICES**

**1a**. In **the** **past 3 months**, have you visited **a long Covid clinic?**

| Yes |  |  |
| --- | --- | --- |
| No |  | (If **no**, go to question 1c) |

**1b.** If **‘Yes’,** how many times in **the past 3 months** did you visit **a long Covid clinic?**

**.……………times**

**1c**. In **the** **past 3 months**, have you visited any **other clinic at a hospital** as an **out-patient?**

| Yes |  |  |
| --- | --- | --- |
| No |  | (If **no**, go to question 2**a**) |

**1d.** If **‘Yes’,** how many times in **the past 3 months** did you visit **a clinic at a hospital?**

**.……………times**

**1e**. For **each** visit to a **clinic at a hospital** please fill in the table below.

| **Visit** | **Reason for visit** |
| --- | --- |
| *Example* | *Appointments to see consultant in neurology or rheumatology* |
| **Clinic Visit 1** |  |
| **Clinic Visit 2** |  |
| **Clinic Visit 3** |  |
| **Clinic Visit 4** |  |
| **Clinic Visit 5** |  |
| **Clinic Visit 6** |  |

X

**2a**. During **the past 3 months**, have you been an inpatient (i.e. **stayed overnight**) in any **hospital/rehabilitation unit** for reasons **related to your long COVID?**

| Yes |  |  |
| --- | --- | --- |
| No |  | (If **no**, go to question 3a) |

*Please* ***tick*** *either Yes or No.*

**2b.** If **‘Yes’,** how many times in **the** **past 3 months** have you been an inpatient for **reasons related to your long COVID?**

**.……………times**

**2c**. For **each** inpatient stay please complete the following information:

| **Inpatient stay** | **Reason for the inpatient stay** | **Number of nights spent in hospital** | **Name of hospital/ rehabilitation unit/** | **Was this an emergency admission following attendance at A&E?** |
| --- | --- | --- | --- | --- |
| *Example* | *Admitted for chest pain and shortness of breath* | *4* | *Morriston Hospital, Swansea* | \| X \| *Yes* \| \| --- \| --- \| \|  \| No \| |
| Inpatient stay 1 |  |  |  | \|  \| Yes \| \| --- \| --- \| \|  \| No \| |
| Inpatient stay 2 |  |  |  | \|  \| Yes \| \| --- \| --- \| \|  \| No \| |
| Inpatient stay 3 |  |  |  | \|  \| Yes \| \| --- \| --- \| \|  \| No \| |

**3a**. In **the** **past 3 months**, have you visited a hospital to receive a **day case procedure** *(i.e. received treatment but you* ***did not*** *spend the night)* for reasons **related to your long COVID?** - *Please only include treatments here. We will ask you for tests and investigations separately in Part 2.*

| Yes |  |  |
| --- | --- | --- |
| No |  | (If **no**, go to question 4**a**) |

*Please* ***tick*** *either Yes or No.*

**3b.** If **‘Yes’,** how many times in **the past 3 months** did you receive a **day case procedure** **related to your long COVID**?

.……………**times**

**3c**. For **each** day case please fill in the table below.

| **Visit** | **Reason for visit** | **Hospital department/ clinic visited** | **Time spent in hospital (hours)** |
| --- | --- | --- | --- |
| *Example* | *I saw a physiotherapist* | *Physiotherapy* | *1.5* |
| Visit 1 |  |  |  |
| Visit 2 |  |  |  |
| Visit 3 |  |  |  |
| Visit 4 |  |  |  |

| Yes |  |  |
| --- | --- | --- |
| No |  | (If **no**, go to question **5a**) |

**4a**. During **the past** **3 months** did you visit any **Accident and Emergency** (A&E or Casualty) for reasons **related to your long COVID** which did **not** lead to an inpatient stay**?**

*Please* ***tick*** *either Yes or No.*

**4b.** If **‘Yes’**, please write the number of times have you attended an A&E department for reasons **related to your long COVID that did not lead to an inpatient admission**?

.……………**times**

**5a**. In **the** **past 3 months** have you used any **community-based health** and **social work** services (including in your own home) for **reasons related to your long COVID (**please **do not** include any contacts which took place in a hospital)**?**

| Yes |  |  |
| --- | --- | --- |
| No |  | (If **no**, go to question **5c**) |

*Please* ***tick*** *either Yes or No.*

**5b.** If ‘**Yes’**, please complete the table:

| **Type of service** | **Have you used this service?**  (tick as appropriate) | **Number of visits/contacts during the last 3 months** |
| --- | --- | --- |
| Telephone/video call with your hospital doctor from your home | Yes No | \|  \|  \| \| --- \| --- \| |
| Telephone/video call with your hospital nurse from your home | Yes No | \|  \|  \| \| --- \| --- \| |
| Appointment with a GP at the GP practice | Yes No | \|  \|  \| \| --- \| --- \| |
| Home visit from a GP | Yes No | \|  \|  \| \| --- \| --- \| |
| Telephone/ video call with a GP | Yes No | \|  \|  \| \| --- \| --- \| |
| Appointment with a GP practice nurse at the GP practice | Yes No | \|  \|  \| \| --- \| --- \| |
| Telephone/video call with a GP practice nurse | Yes No | \|  \|  \| \| --- \| --- \| |
| Home visit from a district/community nurse | Yes No | \|  \|  \| \| --- \| --- \| |
| Attendance at home by a paramedic (999 call) which did not require transfer to A&E (casualty) | Yes No | \|  \|  \| \| --- \| --- \| |
| NHS 111 or NHS Direct Wales telephone call | Yes No | \|  \|  \| \| --- \| --- \| |
| Visit to a community pharmacist for health advice | Yes No | \|  \|  \| \| --- \| --- \| |
| Appointment with an NHS physiotherapist at a health centre/GP practice | Yes No | \|  \|  \| \| --- \| --- \| |
| Appointment with an NHS physiotherapist at your own home | Yes No | \|  \|  \| \| --- \| --- \| |
| Remote consultation with an NHS physiotherapist via computer or phone | Yes No | \|  \|  \| \| --- \| --- \| |
| Appointment with a private physiotherapist | Yes No | \|  \|  \| \| --- \| --- \| |
| Appointment with an NHS occupational therapist at a health centre/GP practice | Yes No | \|  \|  \| \| --- \| --- \| |
| Appointment with an NHS occupational therapist at your own home | Yes No | \|  \|  \| \| --- \| --- \| |
| Remote consultation with an NHS occupational therapist via computer or phone | Yes No | \|  \|  \| \| --- \| --- \| |
| Appointment with a private occupational therapist | Yes No | \|  \|  \| \| --- \| --- \| |
| Appointment with a social worker at a centre/GP practice | Yes No | \|  \|  \| \| --- \| --- \| |
| Home visit with a social worker | Yes No | \|  \|  \| \| --- \| --- \| |
| Telephone/video call with a social worker | Yes No | \|  \|  \| \| --- \| --- \| |
| Appointment with an NHS psychologist | Yes No | \|  \|  \| \| --- \| --- \| |
| Other community-based care, e.g. other therapist**,** day centre/club (please describe):  …………………………………………………….  ……………………………………………………..  ………………………………………………………… | | \|  \|  \| \| --- \| --- \|  \|  \|  \| \| --- \| --- \|  \|  \|  \| \| --- \| --- \| |

**5c. Did you attend a long COVID rehabilitation programme?**

| Yes |  |  |
| --- | --- | --- |
| No |  | (If **no**, go to question **5f**) |

*Please* ***tick*** *either Yes or No.*

**5e. How many sessions of the programme did you attend?**

**_____________sessions**

**5f.** Please record below any private, alternative or complementary health services you have used **and paid for yourself**, in **the past 3 months** for **reasons related to your long COVID** that you have not already recorded.

| **Complementary and alternative care** e.g. osteopath, Reiki, massage therapist, gym membership etc. **(please describe below)** | **What did you pay per visit/contact/month?** | **Number of visits/contacts during the last 3 months** |
| --- | --- | --- |
|  | \| £_____________ \|  \| \| --- \| --- \| \|  \|  \| | \|  \|  \| \| --- \| --- \| |
|  | \| £_____________ \|  \| \| --- \| --- \| \|  \|  \| | \|  \|  \| \| --- \| --- \| |
|  | \| £_____________ \|  \| \| --- \| --- \| \|  \|  \| | \|  \|  \| \| --- \| --- \| |
|  | \| £_____________ \|  \| \| --- \| --- \| \|  \|  \| | \|  \|  \| \| --- \| --- \| |

**Part 2: Tests and Investigations**

**6a**. In **the** **past 3 months** have you have you had any tests or investigations for **reasons related to your long COVID?**

| Yes |  |  |
| --- | --- | --- |
| No |  | (If **no**, go to question **7a**) |

*Please* ***tick*** *either Yes or No.*

**6b.** If ‘**Yes’**, please complete the table:

| **Type of investigation** | **Have you had this investigation?**  (tick as appropriate) | **Number of investigations during the last 3 months** |
| --- | --- | --- |
| Blood test | Yes No | \|  \|  \| \| --- \| --- \| |
| Chest X-ray | Yes No | \|  \|  \| \| --- \| --- \| |
| Lung function test | Yes No | \|  \|  \| \| --- \| --- \| |
| CT scan | Yes No | \|  \|  \| \| --- \| --- \| |
| MRI scan | Yes No | \|  \|  \| \| --- \| --- \| |
| Ultrasound Scan | Yes No | \|  \|  \| \| --- \| --- \| |
| Exercise tolerance test | Yes No | \|  \|  \| \| --- \| --- \| |
| Electrocardiogram (ECG) | Yes No | \|  \|  \| \| --- \| --- \| |
| 24-hour ECG | Yes No | \|  \|  \| \| --- \| --- \| |
| Echocardiogram | Yes No | \|  \|  \| \| --- \| --- \| |
| Electromyogram | Yes No | \|  \|  \| \| --- \| --- \| |
| Blood gases test | Yes No | \|  \|  \| \| --- \| --- \| |
| Sleep apnoea test | Yes No | \|  \|  \| \| --- \| --- \| |
| Other tests or investigations (please describe)  …………………………………………………….  ……………………………………………………..  ………………………………………………………… | | \|  \|  \| \| --- \| --- \|  \|  \|  \| \| --- \| --- \|  \|  \|  \| \| --- \| --- \| |

**6c.** Please record below any private tests or investigation that you **paid for yourself**, in **the past 3 months** for **reasons related to your long COVID** that you have not already recorded.

| **Tests or investigation done privately** **(please describe below)** | **What did you pay per investigation?** | **Number of investigations during the last 3 months** |
| --- | --- | --- |
|  | \| £_____________ \|  \| \| --- \| --- \| \|  \|  \| | \|  \|  \| \| --- \| --- \| |
|  | \| £_____________ \|  \| \| --- \| --- \| \|  \|  \| | \|  \|  \| \| --- \| --- \| |
|  | \| £_____________ \|  \| \| --- \| --- \| \|  \|  \| | \|  \|  \| \| --- \| --- \| |
|  | \| £_____________ \|  \| \| --- \| --- \| \|  \|  \| | \|  \|  \| \| --- \| --- \| |

**Part 3: mental health services**

**7a**. In **the** **past 3 months** have you have you any community mental health services for **reasons related to your long COVID** (please do not include hospital-based services here)**?**

| Yes |  |  |
| --- | --- | --- |
| No |  | (If **no**, go to question **8a**) |

*Please* ***tick*** *either Yes or No.*

**7b.** If ‘**Yes’**, please complete the table:

| **Type of service** | **Did you use this service?**  (tick as appropriate) | **Number of contacts during the last 3 months** |
| --- | --- | --- |
| Counsellor | Yes No | \|  \|  \| \| --- \| --- \| |
| Community psychiatrist | Yes No | \|  \|  \| \| --- \| --- \| |
| Psychologist | Yes No | \|  \|  \| \| --- \| --- \| |
| Community psychiatric nurse | Yes No | \|  \|  \| \| --- \| --- \| |
| Community Mental Health Team member | Yes No | \|  \|  \| \| --- \| --- \| |
| NHS Mental Health helpline | Yes No o | \|  \|  \| \| --- \| --- \| |
| Memory Services | Yes No | \|  \|  \| \| --- \| --- \| |
| Other services used (please describe)  …………………………………………………….  ……………………………………………………..  ………………………………………………………… | | \|  \|  \| \| --- \| --- \|  \|  \|  \| \| --- \| --- \|  \|  \|  \| \| --- \| --- \| |

**7c.** Please record below any private, alternative or complementary mental health services you have used **and paid for yourself**, in **the past 3 months** for **reasons related to your long COVID** that you have not already recorded.

| **Private, complementary and alternative mental health care,** e.g. anxiety course, counsellor etc. **(please describe below)** | **What did you pay per visit/contact?** | **Number of visits/contacts during the last 3 months** |
| --- | --- | --- |
|  | \| £____________ \|  \| \| --- \| --- \| \|  \|  \| | \|  \|  \| \| --- \| --- \| |
|  | \| £_____________ \|  \| \| --- \| --- \| \|  \|  \| | \|  \|  \| \| --- \| --- \| |
|  | \| £_____________ \|  \| \| --- \| --- \| \|  \|  \| | \|  \|  \| \| --- \| --- \| |
|  | \| £_____________ \|  \| \| --- \| --- \| \|  \|  \| | \|  \|  \| \| --- \| --- \| |

**Part 4: TRAVEL TO SERVICES**

**8a.** Please complete the following table relating to your travel to hospital, GP practice and other community services in **the past 3 months**.

| Which service were you travelling to? | **Main form** of transport. (*Tick* ***one*** *category* ***only.*** *If more than one was used, tick the category you used for the* ***longest*** *distance.)* | | If you travelled by **car**, approximately how many **miles** was the return journey? | If you travelled by **car**, how much did you pay for **parking**?  *(****if no charge put 0****)* | If you travelled by **train, bus, taxi or other** how much was the **return fare**? *(****if no charge put 0****)* |
| --- | --- | --- | --- | --- | --- |
| ***GP practice (Inc. visits to practice nurse)*** | Car  Bus  Taxi | Walk  Train  Other | ……….. miles | £…………….. | £…………….. |
| **Other community service**  *(describe below)*:  …………………….  ……………………. | Car  Bus  Taxi | Walk  Train  Other | ……….. miles | £…………….. | £…………….. |
| **Hospital visits** | NHS Ambulance/car  Car  Bus  Taxi | Walk  Train  Other | ……….. miles | £…………….. | £…………….. |

**8b. In the last 3 months,** did you have any additional mobile phone or home phone bills because of accessing services **related to your long COVID** remotely?

| Yes |  |  |
| --- | --- | --- |
| No |  | (If no, go to question 9a) |

*Please* ***tick*** *either Yes or No.*

**8c.** How much money did you spend approximately on phone calls and remote access to services related to your long COVID **in the last 3 months?**

**£………………**

**Part 5: HOME CARE**

**The next question asks about the PAST WEEK.**

**9a.** Are you currently in receipt of a regular care package or home help?

| Yes |  |  |
| --- | --- | --- |
| No |  | (If no, go to question 10a) |

*Please* ***tick*** *either Yes or No.*

**9b.** Have you received regular care or home help **before you started to suffer from long COVID?**

| Yes |  | (If yes, go to question 9d) |
| --- | --- | --- |
| No |  | (If no, go to question 9c) |

*Please* ***tick*** *either Yes or No.*

**9c. Please provide the approximate date when you first started to receive a care package as a consequence of your long COVID:**

**…./…./…….. (DD/MM/YYYY)**

**9d.** Has a Home Care Worker (somebody employed by a Local Authority or an agency) visited you to help you with activities of daily living such as getting in and out of bed, bathing, getting dressed, cleaning, domestic chores, shopping, etc in **the past week** for reasons **relating to your long COVID?**

| Yes |  |  |
| --- | --- | --- |
| No |  | (If no, go to question 12a) |

*Please* ***tick*** *either Yes or No.*

**9e.** If **‘Yes’**, how many times did a Home Care Worker visit you in **the past week**?

.……….........**times**

**9f.** What was the average duration of each visit (in minutes)?

.…….........**minutes**

**9g.** Did you pay for this service?

| Yes |  |  |
| --- | --- | --- |
| No |  | (If no, go to question 12a) |

*Please* ***tick*** *either Yes or No.*

**9h.** If **‘Yes’**, how much did this service cost you in **the** **past week**?

**£**……………..........

**Part 6: TIME OFF WORK AND USUAL ACTIVITIES**

| Yes |  |  |
| --- | --- | --- |
| No |  | (If no, go to question 11a) |

**10a.** Are you currently in paid work or self-employed?

*Please* ***tick*** *either Yes or No.*

**10b.** If **‘Yes’,** please write down the number of hours **per week** you are currently contracted to work or usually work if you are self-employed.

…………… **hours per week**

**10c.** If **‘Yes’**, in **the past 3 months**, approximately how many **days off work** have you taken because of reasons **related to your long COVID** *(please complete days for* ***paid and unpaid leave****, if zero please put 0)***?**

…..…………. **days of PAID LEAVE in past 3 months**

…………. **days of UNPAID LEAVE in past 3 months**

**If you answered 10a-c, please go to question 12a.**

| Yes |  |  |
| --- | --- | --- |
| No |  | (If no, go to question 12a) |

**11a.** Did you stop working (i.e. resigned or retired on ill health) due to your long COVID in the past 3 months?

*Please* ***tick*** *either Yes or No.*

**11b.** When did you stop working (i.e. resigned or retired on ill health) due to your long COVID?

Please provide date: __/__/____ (DD/MM/YYYY)

**12a. In the past 3 months,** have you struggled to pay your bills or afford social activities (e.g. paying for your hobbies or travelling to venues) **due to your long COVID?**

| Yes |  |  |
| --- | --- | --- |
| No |  | (If no, go to question 13a) |

*Please* ***tick*** *either Yes or No.*

12b. If you would like to, please specify which expenses you struggled with because of your long COVID (e.g. rent, utility bills, child care, paying for my hobbies, family days out etc.)?

|  |
| --- |

**12c.** In the past 3 months, did you have to pay for additional child care (e.g. private childminder) because of your long COVID?

| Yes |  |  |
| --- | --- | --- |
| No |  | (If no, go to question 13a) |

*Please* ***tick*** *either Yes or No.*

**If yes, approximately how much money did you spend on additional childcare in the last 3 months?**

**£………………………..**

**13a.** In **the past 3 months,** have you lost time from your normal activities (e.g. caring duties, voluntary work, leisure, sports, hobbies and social events), which you would normally do but cannot do for **reasons related to your long COVID?**

| Yes |  |  |
| --- | --- | --- |
| No |  | (If no, go to question 14a) |

*Please* ***tick*** *either Yes or No.*

**13b.** If **‘Yes’**, in **the** **past week**, approximately how many **hours** have you **lost from normal activities**?

……………… **hours per week**

**If you find it difficult to quantify how much long COVID has impacted your usual activities outside work, please tick the box that most applies:**

| Long COVID hardly affects my usual activities. |  |
| --- | --- |
| I can only do half of what I used to do. |  |
| Long COVID has taken away all my normal activities. |  |

**14a. Do you have a main carer (i.e. helps provide you with regular support with daily activities related to your long COVID such as** getting in and out of bed, bathing, getting dressed, cleaning, domestic chores, shopping**) who is a family member or friend?**

| Yes |  |  |
| --- | --- | --- |
| No |  | (If no, go to question 15a) |

*Please* ***tick*** *either Yes or No.*

**14b.** If ‘**YES’** how many **hours per week** has been spent **caring for you**?

….. hours per week

**Part 7: USE OF MEDICATIONS**

| Yes |  |  |
| --- | --- | --- |
| No |  | (If no, go to question 16a) |

**15a.** Have you used any **non-prescribed** (over the counter/ own purchase) medications, such as paracetamol, creams, gels, herbal remedies, vitamins, etc. in **the past 3 months** for reasons **related to your long COVID?**

*Please* ***tick*** *either Yes or No.*

**15b.** If **‘Yes’**, approximately how much have you spent in the **past 3 months**?

**£**………….........

**16a.** Have you used any medications or preparations **prescribed for you by a GP or consultant** in **the past** **3 months** for reasons **relating to your long COVID?**

| Yes |  |  |
| --- | --- | --- |
| No |  | (If no, go to question 17 at baseline or you are now done at follow-up ) |

*Please* ***tick*** *either Yes or No.*

**16b.** If **‘Yes’**, please report in the tables below the details of any medicines you have been prescribed **for reasons related to long COVID** (if you cannot remember the exact day, please put an approximate date).

| **Name or brand of medicine or preparation** | **Have you been prescribed this medicine in the last 3 months?** (please tick if “yes”) | **What was the strength in mg, mcg, ml, etc.?** (copy strength from bottle/packet) | **Number** (tablets, injections, spoonfuls, etc.) | **Frequency**  *(please tick “per day” or “as required”)* | **Date you started taking the medicine**  *(dd/mm/yy)* | **Date you stopped taking the medicine** *(dd/mm/yy or tick if still taking)* |
| --- | --- | --- | --- | --- | --- | --- |
|  | \|  \| Yes \| \| --- \| --- \| | \|  \|  \|  \| mg \| \| --- \| --- \| --- \| --- \| | \|  \|  \| \| --- \| --- \| | \|  \| Per day \| \| --- \| --- \| \|  \| As required \| | *…./…../.…*  More than 3 months ago | *…./…../.…*   \|  \| Still taking \| \| --- \| --- \| |
|  | \|  \| Yes \| \| --- \| --- \| | \|  \|  \|  \| mg \| \| --- \| --- \| --- \| --- \| | \|  \|  \| \| --- \| --- \| | \|  \| Per day \| \| --- \| --- \| \|  \| As required \| | *…./…../.…* More than six months ago | *…./…../.….*   \|  \| Still taking \| \| --- \| --- \| |
|  | \|  \| Yes \| \| --- \| --- \| | \|  \|  \|  \| mg \| \| --- \| --- \| --- \| --- \| | \|  \|  \| \| --- \| --- \| | \|  \| Per day \| \| --- \| --- \| \|  \| As required \| | *.…/…../.….*  More than six months ago | *…./…../.….*   \|  \| Still taking \| \| --- \| --- \| |
|  | \|  \| Yes \| \| --- \| --- \| | \|  \|  \| mg / \| \| --- \| --- \| --- \|  \|  \|  \|  \| mg \| \| --- \| --- \| --- \| --- \| | \|  \|  \| \| --- \| --- \| | \|  \| Per day \| \| --- \| --- \| \|  \| As required \| | *.…/.…./.….*  More than six months ago | *…./…../.….*   \|  \| Still taking \| \| --- \| --- \| |

| Yes |  |  |
| --- | --- | --- |
| No |  | (If no, go to question 17 at baseline or you are now done at follow-up) |

**16c.** Did you pay for your prescribed medications?

| Yes |  |  |
| --- | --- | --- |
| No |  | (If no, go to question 17 at baseline or you are now done at follow-up) |

**16d.** If **yes,** do you use a pre-payment certificate?

**16e.** If **yes,** please complete:

| 1. The cost was £………………………. |
| --- |
| 1. for …………………… months |

**PART 8: Longer term costs and impact of long COVID**

**17. OPTIONAL:** We recognise this questionnaire above does not accurately capture all the health care and support needs associated with your long COVID because it's restricted to the past 3 months. If you would like us to capture the entire impact of long COVID, please can you use the text box below to provide a general description of your health care and support needs since you developed long COVID:

|  |
| --- |

# APPENDIX 2: SAMPLE TABLES AND FIGURES

These tables are for illustration purposes only and may not reflect the final tables presented in the monograph.

Table A: Costs of the LISTEN intervention

| **INTERVENTION COST**  DRAFT  **Based on x individuals across x groups receiving x intervention across x sites.** | | | | | |
| --- | --- | --- | --- | --- | --- |
|  | **Resource** | **Explanation/Details** | **Unit Cost** | **Resource Usage** | **INTERVENTION TOTAL** |
| Development | (List) |  |  |  |  |
| Training | Training provided by… | *e.g. Training lasts 2 hours plus 30 mins set up. Training delivered once at each site* | *e.g. £60 per hour* | *e.g. 2 hours at each of 10 sites* | *e.g. £1,200* |
|  | Training given to… |  |  |  |  |
|  | Other training resources (List) |  |  |  |  |
| Resources required for delivery of LISTEN intervention | Delivered by… |  |  |  |  |
|  | Other staff resources… |  |  |  |  |
|  | Intervention consumables (list) | *e.g. may include purchased items, training manuals etc* |  |  |  |
|  | … |  |  |  |  |
| Other costs involved in intervention | (List) | *As if provided in standard care. Excludes research costs and any costs that would be incurred by Control.* |  |  |  |
| **TOTAL COST OF LISTEN INTERVENTION** | |  |  |  |  |
| **COST OF LISTEN INTERVENTION PER PARTICIPANT** | |  |  |  |  |

Table B: Use of Health and Social Service – Contacts (example only)

|  | **Arm** | **n** | **Sum** | **Mean** | **SD** | **95% CI of Difference** | **p-value** |
| --- | --- | --- | --- | --- | --- | --- | --- |
| **ASSESSMENT: Baseline/Follow-Up** | | | | | | | |
| **GENERAL PRACTICE AND COMMUNITY NURSING SERVICES** | | | | | | | |
| Number of GP/nurse surgery visits | Control | $x$ | $x$ | $x$ | $x$ | $x$  $(x,x)$ | $x$ |
|  | Intervention | $x$ | $x$ | $x$ | $x$ |  |  |
| Number of GP/nurse home visits | Control | $x$ | $x$ | $x$ | $x$ | $x$  $(x,x)$ | $x$ |
|  | Intervention | $x$ | $x$ | $x$ | $x$ |  |  |
| Number of GP/nurse telephone consultations | Control | $x$ | $x$ | $x$ | $x$ | $x$  $(x,x)$ | $x$ |
|  | Intervention | $x$ | $x$ | $x$ | $x$ |  |  |
| Number of helpline consultations | Control | $x$ | $x$ | $x$ | $x$ | $x$  $(x,x)$ | $x$ |
|  | Intervention | $x$ | $x$ | $x$ | $x$ |  |  |
| **ACCIDENT AND EMERGENCY SERVICES** | | | | | | | |
| Number of Accident and Emergency Unit visits | Control | $x$ | $x$ | $x$ | $x$ | $x$  $(x,x)$ | $x$ |
|  | Intervention | $x$ | $x$ | $x$ | $x$ |  |  |
| Number of ambulance attendances | Control | $x$ | $x$ | $x$ | $x$ | $x$  $(x,x)$ | $x$ |
|  | Intervention | $x$ | $x$ | $x$ | $x$ |  |  |
| Number of admissions from A&E | Control | $x$ | $x$ | $x$ | $x$ | $x$  $(x,x)$ | $x$ |
|  | Intervention | $x$ | $x$ | $x$ | $x$ |  |  |
| Number of unplanned nights spent in hospital | Control | $x$ | $x$ | $x$ | $x$ | $x$  $(x,x)$ | $x$ |
|  | Intervention | $x$ | $x$ | $x$ | $x$ |  |  |
| **HOSPITAL AND OUTPATIENT SERVICES** | | | | | | | |
| Number of outpatient attendances and day cases | Control | $x$ | $x$ | $x$ | $x$ | $x$  $(x,x)$ | $x$ |
|  | Intervention | $x$ | $x$ | $x$ | $x$ |  |  |
| Number of planned inpatient days | Control | $x$ | $x$ | $x$ | $x$ | $x$  $(x,x)$ | $x$ |
|  | Intervention | $x$ | $x$ | $x$ | $x$ |  |  |

DRAFT

| **INVESTIGATIONS** | | | | | | | |
| --- | --- | --- | --- | --- | --- | --- | --- |
| Number of imaging procedures (X-ray, CT, MRI) | Control | $x$ | $x$ | $x$ | $x$ | $x$  $(x,x)$ | $x$ |
|  | Intervention | $x$ | $x$ | $x$ | $x$ |  |  |
| Number of blood tests | Control | $x$ | $x$ | $x$ | $x$ | $x$  $(x,x)$ | $x$ |
|  | Intervention | $x$ | $x$ | $x$ | $x$ |  |  |
| Number of echocardiograms | Control | $x$ | $x$ | $x$ | $x$ | $x$  $(x,x)$ | $x$ |
|  | Intervention | $x$ | $x$ | $x$ | $x$ |  |  |
| Number of exercise tolerance test | Control | $x$ | $x$ | $x$ | $x$ | $x$  $(x,x)$ | $x$ |
|  | Intervention | $x$ | $x$ | $x$ | $x$ |  |  |
| Number of … | Control | $x$ | $x$ | $x$ | $x$ | $x$  $(x,x)$ | $x$ |
|  | Intervention | $x$ | $x$ | $x$ | $x$ |  |  |
| **Other services** | | | | | | | |
| Number mental health service contacts | Control | $x$ | $x$ | $x$ | $x$ | $x$  $(x,x)$ | $x$ |
|  | Intervention | $x$ | $x$ | $x$ | $x$ |  |  |
| Number of social care contacts | Control | $x$ | $x$ | $x$ | $x$ | $x$  $(x,x)$ | $x$ |
|  | Intervention | $x$ | $x$ | $x$ | $x$ |  |  |
| Number of medications | Control | $x$ | $x$ | $x$ | $x$ | $x$  $(x,x)$ | $x$ |
|  | Intervention | $x$ | $x$ | $x$ | $x$ |  |  |
| DRAFT | | | | | | | |

DRAFT

Table C: Health and Social Service Usage Costs by Contact Type (example only)

|  | **Mean (£)**  **(std. dev.)** | **95% CI**  **(£)** | **Difference**  **(95% CI)** | **p-value** |
| --- | --- | --- | --- | --- |
| **GENERAL PRACTICE** | | | | |
| Control  (n= xx) | x  (x) | (x, x) | x  (x, x) | x |
| Intervention  (n= xx) | x  (x) | (x, x) | x  (x, x) | x |
| **HOSPITAL AND OUTPATIENT SERVICES** | | | | |
| Control  (n= xx) | x  (x) | (x, x) | x  (x, x) | x |
| Intervention  (n= xx) | x  (x) | (x, x) | x  (x, x) | x |
| **ACCIDENT AND EMERGENCY SERVICES** | | | | |
| Control  (n= xx) | x  (x) | (x, x) | x  (x, x) | x |
| Intervention  (n= xx) | x  (x) | (x, x) | x  (x, x) | x |
| **INVESTIGATIONS** | | | | |
| Control  (n= xx) | x  (x) | (x, x) | x  (x, x) | x |
| Intervention  (n= xx) | x  (x) | (x, x) | x  (x, x) | x |
| **OTHER CONTACTS** | | | | |
| Control  (n= xx) | x  (x) | (x, x) | x  (x, x) | x |
| Intervention  (n= xx) | x  (x) | (x, x) | x  (x, x) | x |

DRAFT

Table D: Example of how the main economic analysis may be presented

|  | **N** | **Adjusted cost (£)**  **Mean (95% CI)** | **Adjusted QALY**  **Mean (95% CI)** | **Incremental cost (£)**  **(95% CII** | **Incremental QALY**  **(95% CI)** | **ICER**  **(£/QALY)** | **NMB (£) at £20,000/QALY**  **(95% CI)** |
| --- | --- | --- | --- | --- | --- | --- | --- |
| **Primary Analysis** | | | | | | | |
| Intervention |  |  |  |  |  |  |  |
| Usual Care |  |  |  |  |  |  |  |

Table E: Clinical and cost-effectiveness outcomes: cost-consequences analysis

|  | **Intervention** | **Control** | **Difference (95% CI)** | **p-value** |
| --- | --- | --- | --- | --- |
| **Cost impact** | | | | |
| Total implementation costs |  |  |  |  |
| Total costs at 3 months |  |  |  |  |
| Implementation costs per person |  |  |  |  |
| Mean cost at 3 months per patient |  |  |  |  |
| **Health impact** | | | | |
| EQ-5D-5L score at 3 months |  |  |  |  |
| Wellbeing score at 3 months |  |  |  |  |
| Social engagement score at 3 months |  |  |  |  |
| Fatigue at 3 months |  |  |  |  |
| SF-12 scores at 3 months |  |  |  |  |

Table F: Sensitivity Analyses (example only)

DRAFT

|  | **N** | **Adjusted cost (£)**  **Mean (95% CI)** | **Adjusted QALY**  **Mean (95% CI)** | **Incremental cost (£)**  **(95% CII** | **Incremental QALY**  **(95% CI)** | **ICER**  **(£/QALY)** | **NMB (£) at £20,000/QALY**  **(95% CI)** |
| --- | --- | --- | --- | --- | --- | --- | --- |
| **Sensitivity Analysis 1:** | | | | | | | |
| Intervention |  |  |  |  |  |  |  |
| Usual Care |  |  |  |  |  |  |  |
| **Sensitivity Analysis 2:** | | | | | | | |
| Intervention |  |  |  |  |  |  |  |
| Usual Care |  |  |  |  |  |  |  |
| **Sensitivity Analysis 3:** | | | | | | | |
| Intervention |  |  |  |  |  |  |  |
| Usual Care |  |  |  |  |  |  |  |

Figure 1: Cost-Effectiveness Plane

|  | (£)  *Intervention More Costly* | |  |
| --- | --- | --- | --- |
| *Intervention Less Effective* | North-West (NW) Quadrant  Control Dominant  *Intervention more costly and less effective than Control* | North-East (NE) Quadrant  Further Evaluation Required  *Intervention more costly and more effective than Control* | *Intervention More Effective* |
|  | South-West (SW) Quadrant  Further Evaluation Required  *Intervention less costly and less effective than Control* | South-East (SE) Quadrant  Intervention Dominant  *Intervention less costly and more effective than Control* |  |
|  | *Intervention Less Costly* | |  |

*Cost-effectiveness plane will be presented for cost-utility analysis

Figure 2: Cost-Effectiveness Acceptability Curve

**WTP Threshold**

**Probability Intervention Cost Effective**


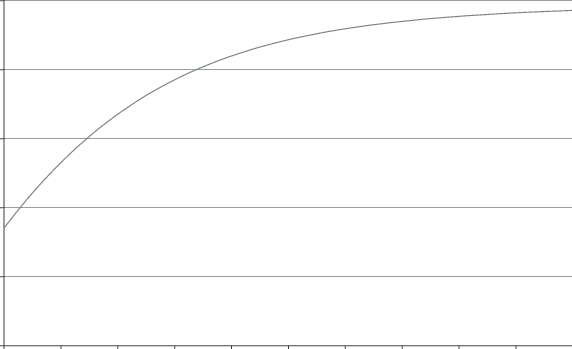


DRAFT

*Cost-effectiveness acceptability curves will be presented for cost-utility analysis
